# Nearly maximal information gain due to time integration in central dogma reactions

**DOI:** 10.1016/j.isci.2023.106767

**Published:** 2023-04-28

**Authors:** Swarnavo Sarkar, Jayan Rammohan

**Affiliations:** 1 National Institute of Standards and Technology, Gaithersburg, MD 20899, USA

**Keywords:** Gene process, Biophysics, Information system model

## Abstract

Living cells process information about their environment through the central dogma processes of transcription and translation, which drive the cellular response to stimuli. Here, we study the transfer of information from environmental input to the transcript and protein expression levels. Evaluation of both experimental and analogous simulation data reveals that transcription and translation are not two simple information channels connected in series. Instead, we demonstrate that the central dogma reactions often create a time-integrating information channel, where the translation channel receives and integrates multiple outputs from the transcription channel. This information channel model of the central dogma provides new information-theoretic selection criteria for the central dogma rate constants. Using the data for four well-studied species we show that their central dogma rate constants achieve information gain because of time integration while also keeping the loss because of stochasticity in translation relatively low (<0.5 bits).

## Highlights

• An information processing model of the central dogma reaction network
• Identifies the maximum protein-level information gain because of time integration
• Reveals the loss in the information gain because of stochasticity during translation
• Naturally occurring central dogma systems achieves nearly maximal information gain

## Graphical abstract

**Figure:**
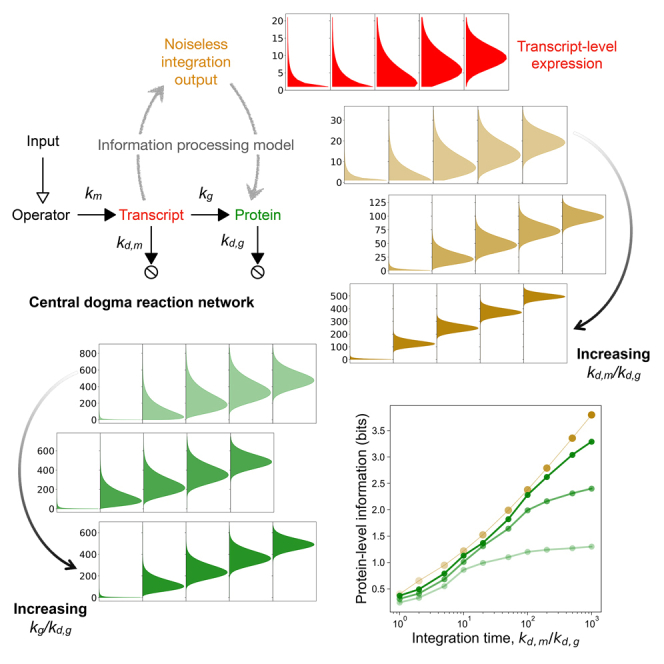

## Introduction

Francis Crick described the central dogma of molecular biology as the unidirectional and sequential flow of information from DNA to RNA to protein through transcription and translation,^1^^,^^2^^,^^3^ which prompts the question: Can we rigorously quantify information transfer in cells from environmental stimuli through transcription and translation? In the past two decades, experimental and computational progress has demonstrated that information transfer can be quantified in biological systems of varying complexity.^4^^,^^5^^,^^6^^,^^7^^,^^8^ However, a quantitative assessment of Crick’s statement requires examining how transcription and translation modulate the information about the environment available in cells.

Quantification of information transfer in biology has been enabled by single-cell measurements.^9^^,^^10^^,^^11^^,^^12^ Previous work has examined information transfer from the environment to either the transcript or the protein expression.^6^^,^^13^^,^^14^^,^^15^ However, those studies have mainly focused on biological networks,^16^ cellular decision making,^17^ or intracellular distribution of information.^18^ There has been a renewed interest in probing the central dogma in recent years.^19^^,^^20^ However, a comprehensive information-theoretic treatment encompassing both transcription and translation, which may explain the naturally occurring central dogma rate constants and inform the design of engineered cellular sensing systems, is still lacking.

Here, we use single-cell measurements and information theory to demonstrate that biology achieves nearly maximal information transfer from the environmental input through translation to the protein expression. We find that the information transfer from the environmental input to the protein expression is higher than the information transfer from the same input to the transcript expression. This contradicts an elementary result from information theory that information should be lost through a simple serial connection of information channels.^21^ To explain this unexpected observation, we develop an information channel model whose properties are functions of the central dogma rate constants. The channel model highlights two distinct properties that affect the information gain during translation: (1) Time integration of the transcript expression, where the amount of signal integration is set by the ratio between the transcript and the protein decay rate constants, and (2) the translation power, i.e., the ratio between the translation rate constant and the protein decay rate constant, or the steady-state mean protein expression per transcript copy, which determines the mean protein expression level. We estimate the *translation loss* as the difference between the maximum possible information gain and the true protein-level information gain. By computing the information gain for multiple species, we demonstrate that the naturally occurring central dogma rate constants result in low translation loss. We also demonstrate that the time period of fluctuations in the environmental input can impose an additional criterion because of which central dogma systems exists in the relatively low integration time regime.

## Results

### Experimental system

**Figure 1:**
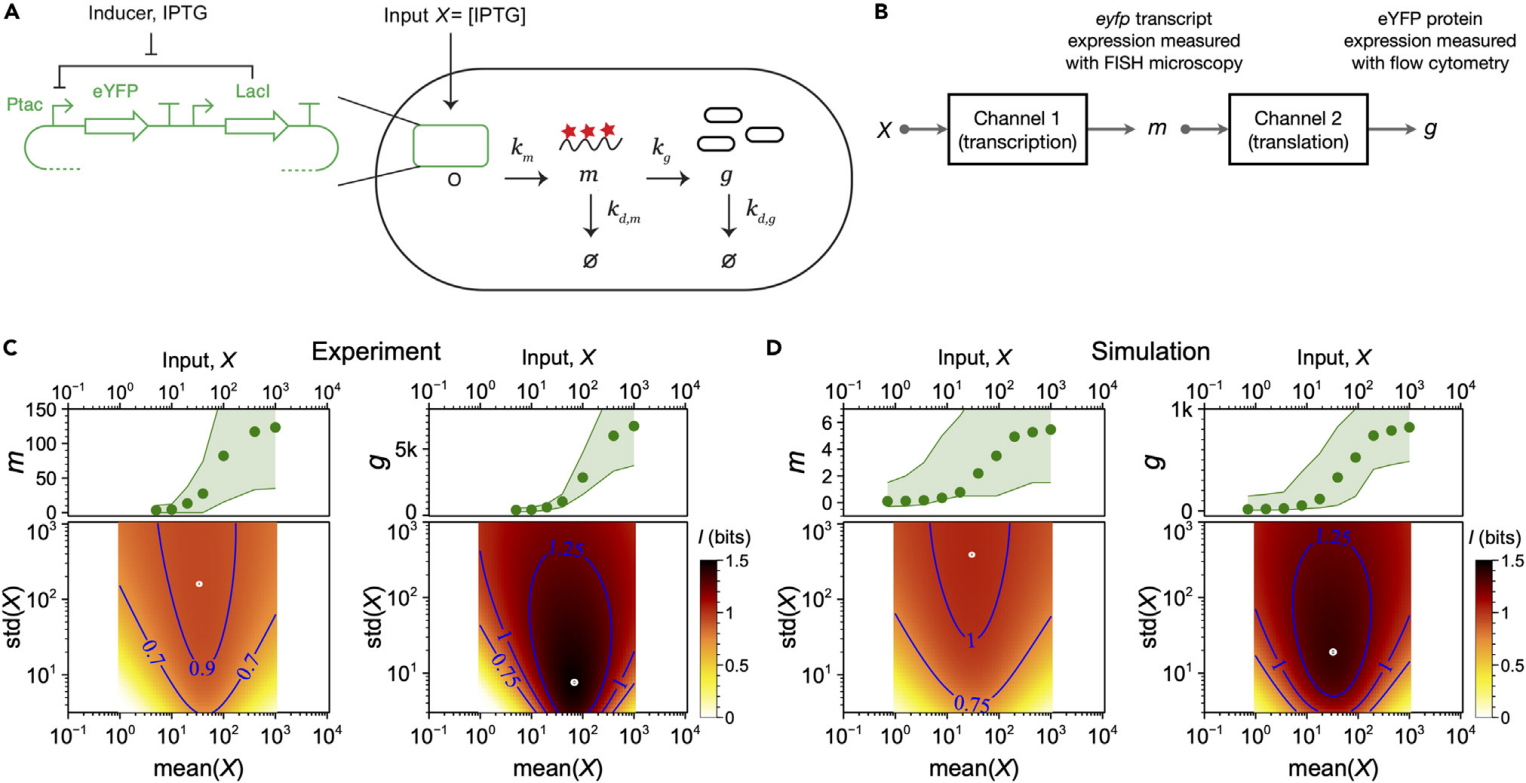
Information flow during transcription and translation (A) The experimental system where IPTG induces the expression of eYFP and we measure both the transcript and the protein expression. The central dogma rate constants are for transcription ($k_{m}$), translation ($k_{g}$), and transcript and protein decays ($k_{d,m},k_{d,g}$).^20^ (B) Sequential channel model of the central dogma process, which receives an input, *X*, and produces transcripts, *m*, and then proteins, *g*, as sequential outputs. (C) Experimental result for the transcript-level mutual information (I(X;m), left), and the protein-level mutual information (I(X;g), right) using data from.^22^ (D) I(X;m) (left) and I(X;g) (right) from simulated expression data using a *lac operon*-based reaction network.^27^ In (C) and (D), the green dots in the top panels are the average expression values. The shaded region bounds the 5%–95% percentiles; the 2D heat maps in the bottom panels show the mutual information values over the space of probability distributions of the input, P(X). The white dots in the heatmap indicate the maximum mutual information. In (C) the transcript value *m* is in RNA counts/cell and the protein value *g* is in molecules of equivalent fluorescein.^63^ In (D) the transcript and protein values are molecules per cell from Gillespie simulations. The maximum mutual information, or the channel capacity, is associated with an optimal input distribution.^21^^,^^24^^,^^27^ The mutual information is higher near the (mean(*X*),std(*X*)) coordinates for the optimal distribution and decreases for input distributions that are away from the optimal one.

To empirically evaluate information transfer in central dogma systems, we used recently reported single-cell measurements of inducible transcript and protein expression for the same gene.^22^^,^^23^ The experimental measurements were made using a system for (2R,3R,4S,5R,6S)-2-(hydroxymethyl)-6-propan-2-ylsulfanyloxane-3,4,5-triol (IPTG)-inducible expression of fluorescent protein in *Escherichia coli*. IPTG serves as the environmental input which regulates the expression of enhanced yellow fluorescent protein (eYFP) (Figure 1A). Single-cell expression of the *eyfp* transcript was measured using single-molecule RNA fluorescence *in situ* hybridization (FISH). Single-cell expression of the eYFP protein was measured using flow cytometry. These measurements of transcript and protein expression within the same system should capture the change in information as it transfers from the environment (*X*) to the transcript expression (*m*) to the protein expression (*g*).

### Quantification of information transfer

To quantify the transfer of information in the experimental system, we consider transcription and translation as information channels, and we determine the mutual information between the environmental input and the transcript expression or the protein expression (method details). Mutual information associated with an information channel depends on both the transition matrix of the channel (P(output|input)) and the probability distribution of input signals,^21^^,^^24^ and the maximum mutual information over all input distributions is the channel capacity.^12^^,^^21^ In this work, both from the experiments and simulations we obtain samples of the *output*, either the transcript or the protein expression level, for a set of values of the *input*. We estimate the conditional distributions for each input, P(output|input), by binning the output samples. Mutual information is obtained for a given input distribution, P(input), by computing the function $I\left(input;output\right)=\sum_{input}P\left(input\right)\sum_{output}P\left(output|input\right)\log_{2}\frac{P\left(output|input\right)}{P\left(output\right)}$.^24^ To compute the channel capacity from the conditional distributions we used the Blahut-Arimoto algorithm,^25^which is an alternating optimization algorithm that has been proven to converge to the maximum mutual information^26^ (STAR Methods). The estimated channel capacity can be biased because of the number of output samples and the number of bins used to construct the conditional distributions. We used existing bootstrapping methods to compute the unbiased estimate of the channel capacity^4^^,^^12^^,^^27^^,^^28^ (STAR Methods). The estimated channel capacity provides the maximum information transfer rate when the input is fluctuating sufficiently slowly for the output to reach the stationary state. The information transfer rate is lower than capacity for fast fluctuations of the input.

If translation acts as a simple information channel that only degrades the information received from the transcription channel (Figure 1B), then we expect the protein-level channel capacity to be lower than the transcript-level channel capacity. Surprisingly, we found the opposite: the experimentally observed protein-level channel capacity (c(X;g)≈ 1.5 bits) is higher than the transcript-level channel capacity (c(X;m)≈ 1.0 bits, Figure 1C). Hence, there exists a gain in information about the input in the translation channel. Moreover, after evaluating both the transcript-level (I(X;m)) and protein-level mutual information (I(X;g)) for a large set of input distributions, we found that the protein expression always contains higher information about the input compared to the transcript expression (Figures 1C, S1 and S2).

The observed information gain in I(X;g) could be because of two artifacts: (1) The transcript expression measurement could be noisier than the protein expression measurement, and (2) there could be unknown biochemical pathways that transfers information directly from the input to the protein expression, bypassing translation. To show that I(X;g)≥I(X;m) is a characteristic property of the central dogma without requiring the above artifacts, we performed Gillespie (kinetic Monte Carlo) simulations of a biochemical reaction network that represents our experimental gene expression system^27^ (method details). The simulated biochemical reaction network contains no unknown reaction pathways and directly provides transcript and protein counts, excluding measurement noise as a factor. Each Gillespie simulation was performed for a fixed value of the input and the resulting protein expression level is an ergodic process.^29^^,^^30^ The mutual information from the Gillespie simulations data were consistent with the experimental results: I(X;g)≥I(X;m) for all input distributions considered (Figures 1D and S2). We will demonstrate that the gain in I(X;g) is because of time integration of the transcript expression, and also depends on the translation power.

**Figure 2:**
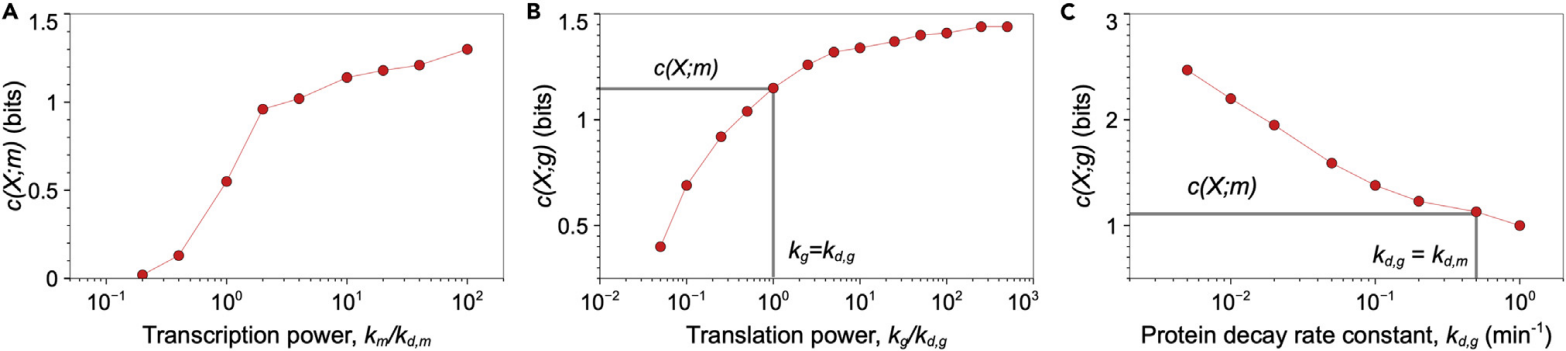
Trends in channel capacity as a function of central dogma rate constants (A) Transcript-level channel capacity for increasing transcription rate constant with fixed transcript decay rate constant, $k_{d,m}=0.5\;\min^{-1}$. The transition in the growth rate of c(X;m) is due toP(m|X) becoming more over-dispersed with increasing $k_{m}/k_{d,m}$ (Figure S3 and method details). (B) Protein-level channel capacity for increasing translation rate constant with fixed protein decay rate constant, $k_{d,g}=0.2\;\min^{-1}$. The black lines highlight that c(X;g)≈c(X;m) when translation power is 1. (C) Protein-level channel capacity for increasing protein decay rate constant with fixed translation power ($k_{g}/k_{d,g}=10^{3}$). The black lines highlight that c(X;g)≈c(X;m) when $k_{d,g}=k_{d,m}$. In (A) to (C) all other rate constants are the same as reported in.^27.^

We used additional Gillespie simulation to explore the impact of the central dogma rate constants on the information transfer. We observed that c(X;m) increases with increasing transcription power (the ratio of the transcription rate constant to the transcript decay rate constant, or the steady-state mean transcript expression, Figure 2A), and c(X;g) increases with increasing translation power (Figure 2B). These trends are similar to the property of simple information channels, e.g., Gaussian or Poisson channels, where channel capacity increases with channel power.^21^^,^^31^^,^^32^ When the translation power is 1, then c(X;g)≈c(X;m), and higher values of translation power appears to increase c(X;g) toward an asymptotic value (Figure 2B). We will demonstrate that this asymptotic value depends on the ratio of the transcript decay rate constant to the protein decay rate constant. Of interest, at fixed transcription and translation powers (i.e., fixed mean protein expression level), c(X;g)*decreases* with increasing protein decay rate constant (Figure 2C). So, the increase in c(X;g) (i.e., the information gained during translation) depends on the transcript and protein decay rate constants and the translation power.

### Channel model

To develop a channel model for the information gain during translation, we start by considering a fundamental result in information theory: Information about the input can only be degraded as it transfers through each information channel. Or, if two information channels are connected in series, then the channel capacity of the combined channel is less than the channel capacity of the first channel.^21^^,^^33^ However, this result is only true for “delayless processing”, in which the second channel only receives one symbol at a time from the first channel to produce a response (i.e., there is no accumulation of the first channel’s output by the second channel^34^^,^^35^). In the context of the central dogma, the transfer of information from transcription to translation is only delayless if the response times for transcription and translation are equal. In general, however, those response times can be different.

To examine how the difference in the two response times causes a gain in c(X;g), we used a generic but sufficient model for transcription and translation that includes the four central dogma rate constants: transcription, transcript decay, translation, and protein decay (Figure 1A and method details). Within the generic model, the stochastic transcript expression is governed by the master equation,

(Equation 1) $$\frac{dP\left(m|O\right)}{dt}=\underset{\text{transcription}}{\underset{︸}{k_{m}OP\left(m-1|O\right)-k_{m}OP\left(m|O\right)}}+\underset{\text{transcript}\;\text{decay}}{\underset{︸}{k_{d,m}\left(m+1\right)P\left(m+1|O\right)-k_{d,m}mP\left(m|O\right)}}$$

where *m* is the number of transcripts, *O* is the promoter state which is either on (O=1) or off (O=0). Both transcription and transcript decay can increase or decrease the occupation probability of the transcript expression level, P(m|O). The stochastic protein expression is governed by the master equation,

(Equation 2) $$\frac{dP\left(g|m\right)}{dt}=\underset{\text{translation}}{\underset{︸}{k_{g}mP\left(g-1|m\right)-k_{g}mP\left(g|m\right)}}+\underset{\text{protein}\;\text{decay}}{\underset{︸}{k_{d,g}\left(g+1\right)P\left(g+1|m\right)-k_{d,g}gP\left(g|m\right)}}$$

where *g* is the number of proteins. From the deterministic ODEs that are obtained by ensemble-averaging the master Equations 1 and 2(method details), the response times for transcription and translation are $1/k_{d,m}$ and $1/k_{d,g}$, respectively. Hence, delayless processing in the central dogma requires $k_{d,m}=k_{d,g}$. However, in general $k_{d,m}\geq k_{d,g}$, typically by a factor of 10.^20^^,^^36^^,^^37^^,^^38^^,^^39^^,^^40^^,^^41^^,^^42^ Consequently, the translation response time is longer than the transcription response time, and the translation channel effectively receives and integrates multiple outputs from the transcription channel. There are existing studies on the emergence of time integration in biochemical reaction networks,^43^^,^^44^ but these earlier studies mainly used signal-to-noise ratio instead of quantifying the information transferred from the environmental input to the biological output. Information transfer quantifies the biochemical work that can be done because of signal transduction.^45^ Therefore, it is important to go beyond a signal-to-noise ratio analysis of central dogma systems and assess the information gain because of time integration.

### Maximum possible information gain during translation

To determine the amount of information lost because of stochasticity in translation, we first calculated the maximum possible information gain because of time integration during translation. In the deterministic model of translation, the protein expression g(t) is the convolution of the transcript trajectory, m(t), with a time integration kernel, $f\left(t\right)=e^{-k_{d,g}t}$ and multiplied by the translation rate constant $k_{g}$ (Figure S4 and method details). In this deterministic model for time integration, $k_{g}$ only scales the convolution output, without increasing the dispersion in the protein expression level, and therefore does not affect the protein-level channel capacity. So, the result of an ideal, noise-free time integration during translation is the hypothetical protein expression level: $g_{\text{ideal}}\left(t\right)\equiv \left(f*m\right)\left(t\right)$. We define the ideal channel capacity $c_{\text{ideal}}\left(T\right)\equiv c\left(X;g_{\text{ideal}}\right)$, as a function of the dimensionless integration time, $T\equiv k_{d,m}/k_{d,g}$. Because the analytical solution to the transcript expression distribution, P(m|X), is known,^6^^,^^46^ we can construct an analytical approximation of $g_{\text{ideal}}$. The number of uncorrelated outputs of the transcription channel received by the translation channel within the latter’s response time is *T*. Therefore, we approximate $g_{\text{ideal}}\approx \sum_{i=1}^{T}m^{\left(i\right)}$, where each $m^{\left(i\right)}$ are independent and identically distributed with the distribution P(m|X). Because the transcript expression level has a negative binomial distribution with NB(r,p) ,^46^ the ideal integration output $g_{\text{ideal}}$ has the distribution NB(rT,p). The ideal channel capacity using $g_{\text{ideal}}∼\text{NB}\left(rT,p\right)$ matches the value from numerical convolution of the transcript expression trajectory (Figure S5). Information gain because of time integration has been previously studied for transcriptional cascades,^47^^,^^48^ but these studies used a Gaussian noise model. We know that transcript expression has a negative binomial distribution,^46^ so our estimate of the maximum possible information gain is likely to be more accurate. But the mechanism of information gain, i.e. time integration, is the same in^47^^,^^48^ and in this work.

**Figure 3:**
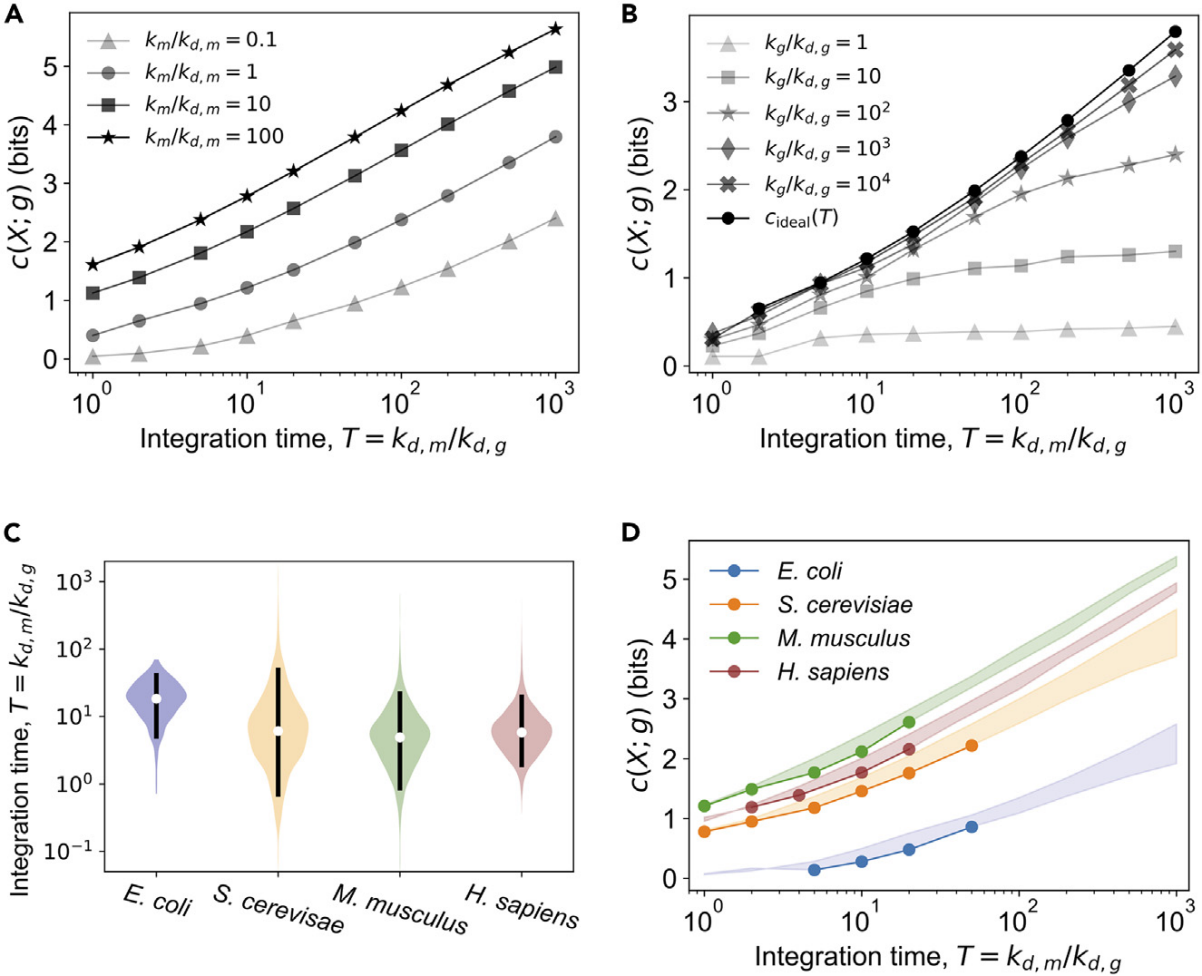
Information gain during translation (A) Ideal information gain curves as a function of the dimensionless integration time, $T\equiv k_{d,m}/k_{d,g}$. $k_{d,m}=0.1\;\min^{-1}$ for all the curves. At T=1, the ideal channel capacity is a function of the transcription power, $k_{m}/k_{d,m}$. For T>1, $c_{\text{ideal}}\left(T\right)$, is generally greater than c(X;m). (B) Protein-level information gain curves for different translation power values, $k_{g}/k_{d,g}$. For the results shown, $k_{m}=k_{d,m}=0.1\;\min^{-1}$, which results in relatively low transcript-level channel capacity, c(X;m)=0.4 bits. The information gain due to time integration is determined by the translation power, $k_{g}/k_{d,g}$, and *T*. (C) Distributions of *T* for four species from literature data. The black bars show the 5%–95% percentile range. The white dots show the median. (D) Ideal and protein-level information gain curves for the four species. The solid lines with filled circles is c(X;g), covering the 5%–95% percentile range of *T*. The shaded regions highlight the translation loss $c_{\text{ideal}}\left(T\right)-c\left(X;g\right)$. The translation loss is shown both within and beyond the naturally occurring range of *T*. For each of the species, the transcription rate constant $k_{m}$ and the transcript decay rate constant $k_{d,m}$ used were near median values in the literature (SI:3B).

We identified the combined effect of $k_{m},k_{d,m}$, and $k_{d,g}$ on $c_{\text{ideal}}\left(T\right)$ using multiple simulated datasets (Figure 3A and method details). First, we determined the transcript expression distribution as a function of $k_{m}$ and $k_{d,m}$, and then we determined $P\left(g_{\text{ideal}}|X\right)$ using the analytical approximation to compute $c_{\text{ideal}}\left(T\right)$ (Figures 3A andS6 and method details). At T=1, $c_{\text{ideal}}\left(T\right)=c\left(X;m\right)$ and at longer integration times, $c_{\text{ideal}}\left(T\right)>c\left(X;m\right)$. Stochasticity in translation reduces the protein-level channel capacity, but this reduction is against $c_{\text{ideal}}\left(T\right)$ and *not* against c(X;m). We only explore the ideal channel capacity for T≥1 in Figure 3A, because most of biology exists in this region.^36^^,^^37^^,^^38^^,^^39^^,^^40^^,^^41^^,^^42^ However, we can estimate the effect of T<1 using the analytical result for the ideal integration output. Because $g_{\text{ideal}}∼\text{NB}\left(rT,p\right)$, the relative standard deviation is proportional to $T^{-0.5}$. So, for T<1, dispersion will increase after translation and subsequently reduce the ideal channel capacity.

### Information lost because of stochasticity in translation

To determine the loss in information after time integration, we used Gillespie simulations of the operator state transition, transcription, transcript decay, translation, and protein decay reactions together (method details) to obtain P(g|X) and then compute c(X;g) for a range of integration times. Both Equations 1 and 2 are birth-death processes and the protein expression level from simulations is an ergodic process.^30^^,^^49^^,^^50^ For each input level we estimated P(g|X) from the stationary state trajectory data from Gillespie simulations. We computed the protein-level information gain curves, c(X;g) vs. *T*, for five values of the translation power, $k_{g}/k_{d,g}$ (method details). At low translation power, there is relatively more noise in the translation output, and c(X;g) is noticeably lower than $c_{\text{ideal}}\left(T\right)$ (Figure 3B). As the translation power increases, the protein-level channel capacity asymptotically approaches the ideal channel capacity.

We observed three features in the information gain curves. First, the protein-level channel capacity increases monotonically with integration time but has a plateau at longer integration times. This plateau is most prominent for translation powers $k_{g}/k_{d,g}\leq 100$ (Figure 3B). Increasing the translation power shifts the plateau region to longer integration times. Second, the translation loss, $c_{\text{ideal}}\left(T\right)-c\left(X;g\right)$, is generally small at low integration times (prominently for $k_{g}/k_{d,g}\geq 100$ when T≤100), but in the plateau region the translation loss increases significantly with *T*. Third, for a fixed integration time the increase in protein-level channel capacity diminishes with increasing translation power, as evident from the small difference in the curves for translation powers $10^{3}$ and $10^{4}$ in Figure 3B. This feature agrees with the previously established result of diminishing gain in the signal-to-noise ratio with increasing translation rate constant.^43^

### Information gain in naturally evolved systems

To estimate the information gain and translation loss for naturally evolved systems, we performed stochastic simulations of the central dogma system, equations Eq. (1) and Equation 2, using typical rate constants for four species from published data: *E. coli, Saccharomyces cerevisiae, Mus musculus*, and *Homo sapiens* (Table S1 and method details).^20^^,^^36^^,^^37^^,^^38^^,^^39^^,^^40^^,^^41^^,^^42^ We used the decay rate constants to determine the distribution of the dimensionless integration time for each species (Figure 3C). The median *T* for *E. coli* is 20 (5%–95% percentile range: 5 to 44). For eukaryotic species, the median *T* is lower: 6 (1–53) for *S. cerevisiae*, 5 (1–24) for *M. musculus*, and 6 (2–21) for *H. sapiens* (Figure 3C and method details). *Bacillus subtilis* appears to have a similar *T* value as *E. coli*.^51^^,^^52^ So, prokaryotes may generally have longer integration times than eukaryotes. However, the dimensionless integration time is relative to the transcription response time, $1/k_{d,m}$. Because $k_{d,m}$ is larger for prokaryotes, the duration of integration in the units of time is still shorter for prokaryotes compared to eukaryotes. Based on the gene ontology enrichment analysis for the *M. musculus* data,^40^ genes with relatively high integration times are associated with dephosphorylation and RNA processing, and genes with relatively low integration times are associated with defense response, homeostasis, and proteolysis — processes that may require faster response times.

We computed $c_{\text{ideal}}\left(T\right)$ and c(X;g) for each species to determine the translation loss (Figure 3D, Tables S2 and S3). Within the typical range of integration times (the 5%–95% percentile range of *T*), the translation loss is less than 0.5 bits (Figure 3D), or the translation power is nearly adequate to transfer the maximum amount of information. Our simulation results demonstrate (Figure 3B), that it is possible to have central dogma rate constants with high translation loss (>1 bit), but we do not observe such rate constants combinations in the literature data. So, low translation loss could be an evolutionary selection criterion for the central dogma rate constants. As a corollary to the observation of low translation loss, the naturally occurring central dogma rate constants do not achieve the plateauing information gain possible for the given translation power. So, maximizing the information gain for a fixed mean protein expression level, by increasing the integration time, is probably not an unconstrained selection criterion. The high translation loss associated with the plateauing protein-level channel capacity can be a constraint against large integration times. Moreover, the observed integration times do not span the full range of low translation loss and stop well below the onset of the plateau region (Figure 3D). Hence, although low translation loss might be an evolutionary selection criterion, it does not appear to be the only criterion.

## Discussion

To understand why naturally evolved systems do not have integration times near and beyond 100, we considered the fluctuation time period of the environmental input. High integration times correspond to longer translation response times, and central dogma systems only operate at channel capacity if the environmental fluctuations are slower than the translation response time (Figures S9 and S10, method details). When the input fluctuation period is less than or comparable to the translation response time, then the translation channel output is correlated with the previous outputs. The correlated outputs decrease the effective channel capacity in a way that is analogous to the reduced capacity of slow-fading information channels.^53^^,^^54^ Using simulated $P\left(g_{\text{ideal}}|X\right)$ data from a fluctuating input protocol, we found that environmental fluctuations have to occur roughly 10 times slower than the integration time for the ideal mutual information $I\left(X;g_{\text{ideal}}\right)$ to be close to capacity $c\left(X;g_{\text{ideal}}\right)$ (Figure S10). Thus, we speculate that naturally evolved central dogma systems use time integration for information gain but remain within the relatively fast translation response times. Earlier work has shown that increasing the number of integration channels in a linear network will keep on increasing the mutual information between the input and the terminal output.^47^ But a linear accumulation of time integration channels also increases the response time of the terminal output, and information transfer is low for fluctuations that are faster than the response time of the output.

The information-theoretic criteria of translation loss and response time emerge as additional constraints for optimal protein expression, which has been previously explored using bioenergetics^55^^,^^56^ and resource allocation.^57^ Of interest, the speed of response has also been identified as a selection criterion using energy and fitness-based analysis.^58^^,^^59^ Because of the connection between thermodynamics and information,^60^ and between information and fitness,^24^^,^^61^ it is likely that these constraints on the optimal protein expression are dependent on each other. Our findings encourage new studies on controlling the fluctuation time period of the input in evolution experiments and observing the subsequent change in the integration time. Evolution experiments in slowly changing environment can reveal if high integration time is a selection criterion and if the high translation loss region is indeed forbidden. Similar alternating environment experiments have been performed to study the impact on fitness.^62^ Even under a matched response and fluctuation time period, we can start with a central dogma system at high translation loss and observe if the mean protein expression increases to reduce the loss. We expect that the energetic and resource constraints will affect the magnitude of reduction in the translation loss.

Information gain because of time integration is directly applicable to gene regulatory networks, where one gene (*input*) controls the expression of another gene (*output*). However, we need additional studies to determine the effect of feedback on ideal channel capacity and translation loss. We had previously analyzed the protein-level information transfer, I(X;g), through synthetic biochemical reaction networks (BRNs) under positive feedback.^27^ We found that positive feedback stabilizes the protein-level mutual information to the same value for a large set of input distributions. The synthetic BRNs in that study^27^ were based on the *lac operon*, where feedback directly controls the operator state. Information gain because of time integration of the transcript expression level is a separate phenomenon. Therefore, we expect information gain during translation to persist even under feedback.

Our information gain model shows that time integration during translation is a general feature that results in a gain of information at the protein expression level. Furthermore, the central dogma rate constants for four well-studied species are typically in a regime where the information loss because of stochasticity in translation is relatively low, suggesting that time integration with low translation loss while avoiding slow response times may be selection criteria for naturally evolved central dogma systems. Our findings also suggest that the typical integration time is lower in eukaryotes than prokaryotes, and decay rate data for additional species could confirm whether this trend is universal. Because the translation loss for natural central dogma systems is small, the ideal channel capacity provides a fast estimate of the protein-level channel capacity that does not require protein expression data, which could be useful for large surveys of biological information transfer.

Beyond transforming the central dogma process from a set of biochemical reactions to an information acquiring and integrating system, these insights are also relevant for engineering synthetic biological information processing systems. Synthetic biology enables the construction of gene expression systems with a wide range of central dogma rate constants. This work presents new quantitative criteria, the translation loss, and the response time relative to the timescale of environmental fluctuations as essential factors to consider for the design of synthetic central dogma systems.

### Limitations of the study

Our model does not include positive or negative feedback during the gene induction process. Quantification of the maximum information gain because of time integration and translation loss in feedback-controlled systems will require additional mathematical and computational analysis. We have used existing techniques to correct the estimated channel capacity for finite-sampling bias and bin-size selection, but to the best of our knowledge there is no proof that these methods completely remove the estimation error. We need the central dogma rate constants data for more species to confirm the universality of our findings about the integration time and translation loss in naturally occurring central dogma systems.

## STAR★Methods

### Key resources table

**Table:** 

REAGENT or RESOURCE	SOURCE	IDENTIFIER
**Deposited data**		

Transcript and protein expression data for the IPTG-inducible gene expression system	Rammohan et al.^22^	https://doi.org/10.1038/s42003-021-02138-6
Reactions and rate constants for the *lac operon*-type network	Sarkar et al.^27^	https://doi.org/10.1038/s42003-020-0901-9
Transcription and translation rate constants for all four species	Hausser et al.^20^	https://doi.org/10.1038/s41467-018-07391-8
Transcript decay rate constant for *E. coli*	Bernstein et al.^36^	https://doi.org/10.1073/pnas.112318199
Protein decay rate constant for *E. coli*	Nagaet al.^37^	https://doi.org/10.1128/mSystems.01296-20
Transcript decay rate constant for *S. cerevisiae*	Eser et al.^38^	https://doi.org/10.1002/msb.134886
Protein decay rate constant for *S. cerevisiae*	Belle et al.^39^	https://doi.org/10.1073/pnas.0605420103
Transcript and protein decay rate constant for *M. musculus*	Schwanhausseret al.^40^	https://doi.org/10.1038/nature10098
Transcript decay rate constant for *H. sapiens*	Friedel et al.^41^	https://doi.org/10.1093/nar/gkp542
Protein decay rate constant for *H. sapiens*	Cambridge et al.^42^	https://doi.org/10.1021/pr101183k

**Software and algorithms**		

Python 3.8	Python Software Foundation	https://www.python.org
Sparse Estimation of Mutual Information Landscapes	SEMIL^64^	https://github.com/usnistgov/InGene

### Resource availability

#### Lead contact

Further information and request for resources should be directed to and will be fulfilled by the lead contact, Swarnavo Sarkar (ss4235@georgetown.edu).

#### Materials availability

This study did not generate new unique reagents.

### Method details

#### Computation of mutual information and channel capacity

To assess the transfer of information from the environmental input to either the transcript or the protein expression levels we computed the mutual information in bits as

(Equation 3) $$I\left(X;g\right)=\sum_{X}P\left(X\right)\sum_{g}P\left(g|X\right)\log_{2}\frac{P\left(g|X\right)}{P\left(g\right)}$$

For the transcript level mutual information, we replace the protein expression level *g* with the transcript expression level *m* in Equation 3. The conditional distributions, P(g|X) or P(m|X), in Equation 3, are empirical distributions of the transcript or protein expression levels for fixed values of the environmental input, either from experiments or from stochastic simulations. The set of transcript or protein expression distributions, P(m|X) or P(g|X), for a set of values of the input, *X*, mathematically defines the information channel for which we compute the mutual information. As seen in Equation 3, mutual information also depends on the input distribution, P(X). The maximum possible mutual information through an information channel for all possible input distributions is the channel capacity, which is calculated by maximizing the mutual information with respect to the input distribution:

(Equation 4) $$c\left(X;g\right)=\max_{P\left(X\right)}I\left(X;g\right)$$

The conditional transcript and gene expression distributions, P(m|X) and P(g|X), respectively, are *input-to-output* transition matrices. The information channel model of X→m or X→g are defined by the respective transition matrices. Throughout this work we computed the channel capacity from the transition matrices using the well-established Blahut-Arimoto algorithm, as described in.^4^^,^^12^^,^^25^^,^^27^ We are providing a short review of the Blahut-Arimoto algorithm, which we have also summarized in an earlier publication.^28^ Given the input distribution p:=P(X) and the channel transition matrix Q:=P(g|X), the mutual information I(p,Q):=I(X;g) is the solution to the following maximization problem,

(Equation 5) $$I\left(p,Q\right)=\max_{R}J\left(p,Q,R\right)=\max_{R}\sum_{j}\sum_{k}p_{j}Q_{k|j}\;\log_{2}\frac{R_{j|k}}{p_{j}}$$

where *R* is a variable *output-to-input* transition matrix or P(input|output). From Equations 3 and 5, the maximum information transfer or the channel capacity is the solution to the double maximization problem,

(Equation 6) $$c\left(X;g\right)=\max_{p}\max_{R}J\left(p,Q,R\right)$$

The Blahut-Arimoto algorithm to compute the channel capacity for a given input-to-output transition matrix, Q, is built using two properties.

1. For a fixed input distribution, *p*, the trial output-to-input transition matrix that maximizes J(p,Q,R) is

(Equation 7) $$R_{j|k}^{*}=\frac{p_{j}Q_{k|j}}{\sum_{j}p_{j}Q_{k|j}}$$

2. For a fixed output-to-input transition matrix, the input distribution that maximizes J(p,Q,R) is

(Equation 8) $$p_{j}=\frac{\exp\;\left(\sum_{k}Q_{k|j}\;\log_{2}R_{j|k}\right)}{\sum_{j}\;\exp\;\left(\sum_{k}Q_{k|j}\;\log_{2}R_{j|k}\right)}$$

The Blahut-Arimoto algorithm is an iterative method that uses Equations 7 and 8 to compute the input distribution that solves the maximization problem in Equation 4.

The channel transition matrix *Q* is obtained by binning the samples of the protein expression level for a fixed input value. The estimated channel capacity can be sensitive to the choice of bin size. If the bins are too coarse, then the channel capacity is underestimated. If the bins are too fine, producing combed conditional distributions, then the channel capacity will be overestimated due to finite-sampling bias.^4^^,^^12^ We corrected for finite-sampling bias as described in.^4^^,^^27^^,^^28^ Briefly, this procedure consists of sampling fractions of multiple sizes of the data, and multiple replicates for each fraction. The channel capacity values for all the fractional samples are computed, which provides a set of biased channel capacity values as a function of the inverse sample size. The unbiased channel capacity is then obtained by extrapolating to the infinite samples limit. This process of estimating the unbiased channel capacity is repeated for increasing number of bins to identify the number of bins that are large enough to capture the variance but not too large to produce combed distributions that can overestimate the channel capacity.^4^ The mutual information landscapes shown in Figures 1C and 1D were computed from the transcript and the protein expression distributions, P(m|X) and P(g|X), respectively, using Sparse Estimation of Mutual Information Landscapes (SEMIL) as described in.^27^

#### Transcript and protein-level mutual information landscapes for biological replicates

To check the reproducibility of the transcript and the protein-level mutual information values we computed the mutual information landscapes with data from 3 biological replicates of the inducible gene expression system.^23^ As mentioned in the main text, the transcript-level expression was measured using microscopy of FISH probes, and the protein-level expression was measured using flow cytometry.^22^ The transcript and the protein-level mutual information landscapes for the 3 replicates are shown in Figure S1. The landscapes show the mutual information values across a space of input distributions, P(X), where the distributions are identified using the mean and the standard deviation of P(X).

#### Simulated inducible gene expression system

To check that the central dogma reactions, consisting only of sequential transcription and translation, can cause the gain in the protein-level mutual information, we computed the mutual information landscapes for a simulated reaction network that is analogous to our experimental system. This reaction network was developed from a previously-published model of the *lac operon*,^27^^,^^29^ consisting of the same set of reactions involving the input (IPTG) and the operator as present in the *lac operon* but without the positive feedback due to the *lacY* gene, as our experimental system does not contain feedback. We used Gillespie simulations to compute the transcript and the protein expression levels for this model reaction network. We used exactly the same set of reactions and rate constants shown in the SI of^27^ in Tables 4 and 5, respectively. We subsequently used the transcript and the protein expression data to compute the mutual information landscapes as described in.^27^ The transcript and the protein-level mutual information landscapes for the simulated inducible gene expression system are shown in Figure 1D of the main text.

#### Difference in the transcript and protein-level mutual information

To show that the gain in the protein-level mutual information exists for all input distributions, we present the difference between the protein-level and transcript-level mutual information landscapes in Figure S2. The protein-level mutual information is higher than the transcript-level mutual information for all the input distributions in the landscape, so the gain in protein-level mutual information is not confined to the input probability distribution that causes maximum information transfer, but is most likely true for all input distributions.

#### Generic stochastic model of transcription and translation

To generate the stochastic transcript and protein expression data for a generic central dogma system, we considered that the information transfer from the environment to the protein expression level occurs through the following three processes.

1. The operator state switching between active (O=1) and inactive (O=0).

(Equation 9) $$\left(O=1\right)\overset{k_{ON}}{\underset{k_{OFF}}{⇌}}\left(O=0\right)$$

2. Transcription and transcript decay.

(Equation 10) $$\begin{array}{c} \left(O=1\right)\overset{k_{m}}{\to }\left(O=1\right)+m\\ m\overset{k_{d,m}}{\to }\emptyset \end{array}$$

3. Translation and protein decay.

(Equation 11) $$\begin{array}{c} m\overset{k_{g}}{\to }m+g\\ g\overset{k_{d,g}}{\to }\emptyset \end{array}$$

The set of processes, (9)-(11), is a sufficient but minimal model of the central dogma system. The interaction of the environmental input with the operator is condensed into the rate constants, $k_{ON}$ and $k_{OFF}$. In this work, we consider the following form for the $k_{ON}$ and $k_{OFF}$ rate constants

(Equation 12) $$\begin{array}{l} k_{ON}=\alpha \left(\left(1-l\right)X+l\right)\\ k_{OFF}=\alpha \left(1-l\right)\left(1-X\right) \end{array}$$

where 0≤X≤1 is the environmental input value. *l* is the leakiness, or $l=k_{ON}/\left(k_{ON}+k_{OFF}\right)$ when X=0, which determines the leaky transcription in the absence of the environmental input. α controls the rate or the frequency of switching between active and inactive operator states, as $k_{ON}+k_{OFF}=\alpha$ independent of the input value *X*. The transcript and the protein-level channel capacity depends only on the expression distributions, P(m|X) and P(g|X), respectively. The shape of mean dose-response curves, ⟨m⟩-vs-*X* or ⟨g⟩-vs-*X*, does not change the channel capacity, as long as the distributions P(m|X) and P(g|X) remain unchanged.

To explain the transition in the transcript-level channel capacity observed in Figure 2A of the main text, we present a parametric simulation study on the growth in the transcript-level channel capacity, c(X;m), as a function of the transcription power, $k_{m}/k_{d,m}$ (Figure S3). We computed c(X;m)-vs-$k_{m}/k_{d,m}$, for a fixed transcript decay rate constant, $k_{d,m}=0.5\;\min^{-1}$ and three values of the frequency parameter $\alpha =\left\{0.1,1,10\right\}\;\min^{-1}$. We determined the transcript distributions P(m|X) as a function of $k_{m},k_{d,m},k_{ON}$, and $k_{OFF}$ using the analytical result for transcript expression distribution,^46^which we subsequently used to compute c(X;m). We observed that when α is comparable to or lesser than $k_{d,m}$, then there is a sharp decrease in the rate at whichc(X;m) increases with $k_{m}/k_{d,m}$ (Figure S3). This transition occurs because at low $k_{m}/k_{d,m}$, the transcript expression distribution P(m|X) is more Poissonian, Fano factor ≈1, but becomes increasingly more over-dispersed, Fano factor >1, for higher $k_{m}/k_{d,m}$.^6^ When $\alpha \gg k_{d,m}$, then the transcript expression distribution remains close to Poissonian for a higher range of transcription powers. Therefore, we do not observe a change in the growth rate of c(X;m) for $\alpha =10\;\min^{-1}$ in Figure S3. The transition will still occur for $\alpha =10\;\min^{-1}$, but at a higher value of $k_{m}/k_{d,m}$.

#### Master equations

The master equation for the active state of the operator is

(Equation 13) $$\frac{dP\left(O=1\right)}{dt}=k_{ON}P\left(O=0\right)-k_{OFF}P\left(O=1\right)$$

Since the operator state can be either active or inactive at a time, the master equation for the inactive state of the operator is similar to Equation 13, with the right hand side multiplied by −1, *i.e.*,

(Equation 14) $$\frac{dP\left(O=0\right)}{dt}=k_{OFF}P\left(O=1\right)-k_{ON}P\left(O=0\right)$$

The master equation for the transcript expression level (or transcript copy number), *m*, is

(Equation 15) $$\frac{dP\left(m|O\right)}{dt}=k_{m}OP\left(m-1|O\right)+k_{d,m}\left(m+1\right)P\left(m+1|O\right)-k_{m}OP\left(m|O\right)-k_{d,m}mP\left(m|O\right)$$

The master equation for the protein expression level (or protein copy number), *g*, for a fixed valued of transcript expression, *m*, is

(Equation 16) $$\frac{dP\left(g|m\right)}{dt}=k_{g}mP\left(g-1|m\right)+k_{d,g}\left(g+1\right)P\left(g+1|m\right)-k_{g}mP\left(g|m\right)-k_{d,g}gP\left(g|m\right)$$

Both Equations 15 and 16 are one-step master equations.

#### Governing equations for mean transcript and protein expression levels

The governing equation for the ensemble-averaged mean transcript expression level, ⟨m|O⟩, for a fixed operator state *O* is obtained by multiplying the transcription master Equation 15 with the transcript expression and summing over all possible values as

(Equation 17) $$\frac{d\left\langle m|O\right\rangle }{dt}=\frac{d\sum_{m=0}^{\infty }mP\left(m|O\right)}{dt}=k_{m}O\sum_{m=0}^{\infty }mP\left(m-1|O\right)+k_{d,m}\sum_{m=0}^{\infty }m\left(m+1\right)P\left(m+1|O\right)-k_{m}O\sum_{m=0}^{\infty }mP\left(m|O\right)-k_{d,m}\sum_{m=0}^{\infty }m^{2}P\left(m|O\right)$$

(Equation 18) $$\frac{d\left\langle m|O\right\rangle }{dt}=k_{m}O\sum_{m=0}^{\infty }mP\left(m-1|O\right)+k_{d,m}\sum_{m=0}^{\infty }m\left(m+1\right)P\left(m+1|O\right)-k_{m}O\left\langle m|O\right\rangle -k_{d,m}\left\langle m^{2}|O\right\rangle =k_{m}O\sum_{m=0}^{\infty }\left(m+1\right)P\left(m|O\right)+k_{d,m}\sum_{m=0}^{\infty }m\left(m-1\right)P\left(m|O\right)-k_{m}O\left\langle m|O\right\rangle -k_{d,m}\left\langle m^{2}|O\right\rangle =k_{m}O-k_{d,m}\left\langle m|O\right\rangle$$

The solution to Equation 18 is

(Equation 19) $$\left\langle m|O\right\rangle \left(t\right)=\frac{k_{m}O}{k_{d,m}}\left(1-e^{-k_{d,m}t}\right)$$

which has a relaxation time constant $\frac{1}{k_{d,m}}$.

The governing equation for the ensemble-averaged mean protein expression level, ⟨g|m⟩ for a fixed transcript expression level is obtained from the translation master equation as

(Equation 20) $$\frac{d\left\langle g|m\right\rangle }{dt}=\frac{d\sum_{g=0}^{\infty }\;gP\left(g|m\right)}{dt}=k_{g}m\sum_{g=0}^{\infty }gP\left(g-1|m\right)+k_{d,g}\sum_{g=0}^{\infty }g\left(g+1\right)P\left(g+1|m\right)-k_{g}m\sum_{g=0}^{\infty }gP\left(g|m\right)-k_{d,g}\sum_{g=0}^{\infty }g^{2}P\left(g|m\right)$$

(Equation 21) $$\frac{d\left\langle g|m\right\rangle }{dt}=k_{g}m\sum_{g=0}^{\infty }gP\left(g-1|m\right)+k_{d,g}\sum_{g=0}^{\infty }g\left(g+1\right)P\left(g+1|m\right)-k_{g}m\left\langle g|m\right\rangle -k_{d,g}\left\langle g^{2}|m\right\rangle =k_{g}m\sum_{g=0}^{\infty }\left(g+1\right)P\left(g|m\right)+k_{d,g}\sum_{g=0}^{\infty }g\left(g-1\right)P\left(g|m\right)-k_{g}m\left\langle g|m\right\rangle -k_{d,g}\left\langle g^{2}|m\right\rangle =k_{g}m-k_{d,g}\left\langle g|m\right\rangle$$

The solution to Equation 21 is

(Equation 22) $$\left\langle g|m\right\rangle \left(t\right)=\frac{k_{g}m}{k_{d,g}}\left(1-e^{-k_{d,g}t}\right)$$

which has a relaxation time constant $\frac{1}{k_{d,g}}$.

#### Translation output for a time-dependent transcript expression

Equation 22 is the average protein expression level for a fixed transcript expression level. By ensemble-averaging the Equation 21 with the transcript expression distribution P(m), we obtain the familiar deterministic ODE for translation as

(Equation 23) $$\frac{d\left\langle g\right\rangle }{dt}=k_{g}\left\langle m\right\rangle -k_{d,g}\left\langle g\right\rangle$$

where both ⟨g⟩ and ⟨m⟩ are time-dependent. We can obtain the solution for a time-dependent average transcript expression level ⟨m⟩(t) using Laplace transform. Let the Laplace transform of ⟨m⟩(t) be M(s) and of ⟨g⟩(t) be G(s), then the Laplace transform of Equation 23 is

(Equation 24) $$sG\left(s\right)=k_{g}M\left(s\right)-k_{d,g}G\left(s\right)$$

with the initial condition $\left\langle g\right\rangle \left(0^{-}\right)=0$. Therefore,

(Equation 25) $$\begin{array}{l} G\left(s\right)=M\left(s\right)\frac{k_{g}}{s+k_{d,g}}\\ \left\langle g\right\rangle \left(t\right)=k_{g}\int_{0}^{t}\left\langle m\right\rangle \left(\tau \right)e^{-k_{d,g}\left(t-\tau \right)}\;d\tau \end{array}$$

#### Deterministic integration approximation for the translation output

Equation 25 is the deterministic model of translation, which neglects the stochasticity associated with the translation and the protein decay processes. We use the convolution operator from Equation 25 to define the output of the deterministic integration of a *stochastic* transcript expression trajectory as

(Equation 26) $$g_{\text{ideal}}\left(t\right)=\left(f*m\right)\left(t\right)=\int_{0}^{t}m\left(\tau \right)e^{-k_{d,g}\left(t-\tau \right)}\;d\tau$$

The convolution kernel *f* accounts for the time integration of the stochastic transcript expression that occurs due to the response time of the translation process, $1/k_{d,g}$. We omit the translation rate constant $k_{g}$ from the operator in Equation 25, because it only scales the output of the convolution without introducing more stochasticity to the output, which is necessary to have any impact on the information transfer. Example of the convolution output, which we have named the *ideal integration output*, as a function of the ratio $k_{d,m}/k_{d,g}$ is shown in Figure S4.

Using Equation 26 we transform m(t) to $g_{\text{ideal}}\left(t\right)$. From a transcript trajectory we obtain the transcript expression distribution, P(m|X), and from the ideal integration output trajectory we obtain the distribution, $P\left(g_{\text{ideal}}|X\right)$. The channel capacity of the integrated output $c\left(X;g_{\text{ideal}}\right)$ is a function of, $k_{d,g}$, or more specifically of the ratio $k_{d,m}/k_{d,g}$. $c\left(X;g_{\text{ideal}}\right)$ as a function of the integration time $T=k_{d,m}/k_{d,g}$ is the information gain due to deterministic time integration during the translation process.

We constructed an analytical approximation of the ideal integration output of the transcript expression. We approximated that after every interval of the transcription response time, $1/k_{d,m}$, the transcript expression is represented using independent and identically distributed random variables, all with the distribution P(m|X). Since the total number of intervals of $1/k_{d,m}$ during the translation response time is $T=k_{d,m}/k_{d,g}$, we define the ideal integration output as

(Equation 27) $$g_{\text{ideal}}^{\left(\text{analyt}.\right)}=\sum_{i=1}^{T}m^{\left(i\right)}$$

On the basis of Equation 27, if the probability generating function of the transcript expression distribution is, G(z;m), then the probability generating function of $g_{\text{ideal}}^{\left(\text{analyt}.\right)}$ is $G{\left(z;m\right)}^{T}$. The transcript expression has either a negative binomial distribution (NB) or a Poisson distribution (Pois) depending on the Fano factor $\frac{\sigma^{2}\left(m\right)}{\left\langle m\right\rangle }$^6^. If m∼NB(r,p), then $g_{\text{ideal}}^{\left(\text{analyt}.\right)}∼\text{NB}\left(rT,p\right)$, where *r* is the number of failures and *p* is the failure probability of the transcript expression distribution. Similarly, if m∼Pois(λ), then $g_{\text{ideal}}^{\left(\text{analyt}.\right)}∼\text{Pois}\left(\lambda T\right)$, where λ is average transcript expression level. Although, *T* is an integer in Equation 27, we extend the use to real values while determining the negative binomial or Poisson distribution of the integrated output. The parameters for the transcript expression were calculated from $k_{ON},k_{OFF},k_{m}$, and $k_{d,m}$ using the method described in,^6^ with

(Equation 28) $$\begin{array}{l} \left\langle m\right\rangle =\frac{k_{ON}}{k_{ON}+k_{OFF}}\frac{k_{m}}{k_{d,m}}\\ b=\frac{\sigma^{2}}{\left\langle m\right\rangle }=1+\frac{k_{d,m}k_{OFF}\left\langle m\right\rangle }{k_{ON}\left(k_{ON}+k_{OFF}+k_{d,m}\right)} \end{array}$$

where $k_{ON}$ and $k_{OFF}$ were calculated for each *X* from Equation 12. When b>1, then the transcript expression distribution is NB(r,p), with p=(b−1)/b, and r=⟨m⟩/(b−1). When b=1, then transcript expression distribution is Pois(λ) with λ=⟨m⟩.

#### Validation of the analytical approximation for ideal channel capacity

To validate that the approximation, $g_{\text{ideal}}^{\left(\text{analyt}.\right)}$, accurately estimates the ideal channel capacity, $c\left(X;g_{\text{ideal}}\right)$, we simulated a central dogma system with parameters, $k_{m}=k_{d,m}=0.5\;\min^{-1}$, leakiness l=0.01, frequency parameter $\alpha =1.0\;\min^{-1}$, and with the input values X={0.0,0.1,0.2,0.3,0.4,0.5,0.6,0.7,0.8,0.9,1}. We selected a set of protein decay rate constants, $k_{d,g}=\left\{0.5,0.2,0.1,0.05,0.02,0.01,0.005,0.002,0.001,0.0005,0.0002\right\}\min^{-1}$, which covers the range of integration times as T={1,2.5,5,10,25,50,100,250,500,1000,2500}. For each *X* we simulated the central dogma system up to transcription, Equations 1, 2, 3, 4, 5, 6, 7, 8, 9, 10, 11, 12, and 13, to obtain a stochastic transcript expression trajectory, m(t). Then we obtained the time-integrated trajectory as $g_{\text{ideal}}\left(t\right)$ using (26), which was then used to compute the channel capacity $c\left(X;g_{\text{ideal}}\right)$. At the same time we determined P(m|X) using $k_{ON},k_{OFF},k_{m}$, and $k_{d,m}$ as described in.^6^ We then computed the probability distribution of $g_{\text{ideal}}^{\left(\text{analyt}.\right)}$ using (27) and subsequently determined $c\left(X;g_{\text{ideal}}^{\left(\text{analyt}.\right)}\right)$.

We performed two numerical case studies to check if $c\left(X;g_{\text{ideal}}\right)=c\left(X;g_{\text{ideal}}^{\left(\text{analyt}.\right)}\right)$. In the first case, we determined the transcript stochastic trajectory for $10^{4},10^{5},\text{and}\;10^{6}$ samples at an interval of $1/k_{d,m}$, and convoluted them with the kernel f(t)which was represented in the time domain $\left[0,4/k_{d,g}\right]$ with values at interval of $1/k_{d,m}$. We found that $c\left(X;g_{\text{ideal}}\right)=c\left(X;g_{\text{ideal}}^{\left(\text{analyt}.\right)}\right)$ within 0.1 bits for all the 3 sizes of trajectory data across the entire range of integration time Figure S5A. In the second case, we chose the transcript trajectories with $10^{5}$ samples and convoluted with 4 representations of f(t), with increasing time domains $\left[0,1/k_{d,g}\right],\left[0,2/k_{d,g}\right],\left[0,4/k_{d,g}\right]$, and $\left[0,8/k_{d,g}\right]$. We found that when the time domain is $\left[0,4/k_{d,g}\right]$ or larger, then the ideal channel capacity from the numerical convolution matches with the analytical approximation (within 0.1 bits), Figure S5B. Based on the results in Figure S5, we conclude that for a sufficiently large transcript expression trajectory and convolution kernel domain $c\left(X;g_{\text{ideal}}\right)=c\left(X;g_{\text{ideal}}^{\left(\text{analyt}.\right)}\right)$.

#### Effect of number of input levels on the estimate of $c_{\text{ideal}}\left(T\right)$

The estimate of the ideal channel capacity is bounded from above by $\log_{2}\left|X\right|$, where |X| is the number of input values *X* for which we have the distributions $P\left(g_{\text{ideal}}|X\right)$, either from numerical convolution or analytical approximation. Since we used 11 values of *X* for Figure S5, all the information gain curves peak at $\log_{2}\;11\approx 3.5$ bits. However, the correct value of $c_{\text{ideal}}\left(T\right)$ is not bounded by the number of input values. To remove the underestimation of $c_{\text{ideal}}\left(T\right)$due to the number of input values, we systemically increased the number of input levels as |X|∈{4,8,16,32,64,128} for X∈[0,1], which increases the entropy of the input as H(X)∈{2,3,4,5,6,7} bits, respectively. For each set of input values, we computed $c_{\text{ideal}}\left(T\right)$which is shown in Figure S6. In the range of integration time, T∈[1,2500], 64 input levels is adequate to accurately estimate $c_{\text{ideal}}\left(T\right)$, becaus increasing the number of input values to 128 produces no noticeable difference (less than 0.04 bits). It is necessary to check the convergence in the estimated $c_{\text{ideal}}\left(T\right)$ for increasing number of input levels |X|. Once we is sufficiently high we will avoid underestimation of $c_{\text{ideal}}\left(T\right)$, and also avoid the underestimation of the protein-level channel capacity c(X;g), because $c\left(X;g\right)<c_{\text{ideal}}\left(T\right)$.

#### Stochastic simulations of central dogma master equations

##### Parameters for the generic information gain curves

To produce the information gain curves in Figures 3A and 3B, the following parameters were chosen to model the central dogma system, leakiness, l=0.01, frequency parameter $\alpha =1.0\;\min^{-1}$, and input values X∈[0,1], which determines $k_{ON}$ and $k_{OFF}$ using Equation 12. For the ideal information gain curves, $c_{\text{ideal}}\left(T\right)$, in Figure 3A, the transcript decay rate was $k_{d,m}=0.1\;\min^{-1}$, and the transcription rate constant $k_{m}=\left\{0.01,0.1,1.0,10.0\right\}\min^{-1}$ was determined using the transcription power $k_{m}/k_{d,m}$ values shown in Figure 3A. The set of integration times was T={1,2,5,10,20,50,100,200,500,1000}. The distribution of the ideal integration output was obtained using 27, which was then used to compute the ideal channel capacity, $c_{\text{ideal}}\left(T\right)$.

To produce the protein-level information gain curves in Figure 3B the following central dogma rate constants were used: $k_{m}=k_{d,m}=0.1\;\min^{-1}$, close to the median transcript decay rate constants for *E. coli* and *S. cerevisiae*. The protein decay rate was kd,g = {0.1, 0.05, 0.02, 0.01, 0.005, 0.002, 0.001, 0.0005, 0.0002 $,0.0001\}\;\min^{-1}$, and the translation rate constant was determined using the value of the translation power, $k_{g}/k_{d,g}=\left\{1,10,10^{2},10^{3},10^{4}\right\}$. For integration time T≤20, we chose 4 bits sized input or 16 uniformly-spaced values of *X* in [0,1]. For higher integration times, we chose 64 uniformly-spaced values of *X* in [0,1]. We performed Gillespie simulation of the operator state change and the central dogma reactions, transcription, transcript decay, translation, and protein decay together (9), (10), and (11) to obtain the protein expression distribution for each input value, P(g|X), which was then used to compute the channel capacity, c(X;g). Each stochastic simulation was run for a duration of $10^{6}\;\min$ and $10^{5}$ samples of the protein expression level *g* was obtained at a time interval of 10min. The conditional distributions P(g|X) were obtained from empirical distributions by binning the protein expression data. The number of bins, $n_{b}$, were 8 for T=1 and 32 for T=1000, and a linearly increasing function of logT for the intermediate integration times.

##### Parameters for the information gain curves for the four species

The transcription and the translation rate constants were obtained from sources reported in the key resources table. We determined the distribution of the dimensionless integration time, $T=k_{d,m}/k_{d,g}$, from the paired transcript and protein decay rate constants for each species (Figure S7). The distribution of integration times are shown as violin plots in Figure 3C. For *E. coli*, the effective protein decay rate was determined using a doubling time of 2 h.^66^ The 5%–95% confidence interval was computed using numpy’s percentile function in python, which ranks the samples and determines the percentile value using linear interpolation. The percentile values for the integration time, *T*, reported in the main text have been rounded off to the nearest integer. The violin plots in the main text were determined using matplotlib library’s violinplot function with the ‘scott’ method for density estimate bandwidth.

To determine the ideal and the protein-level information gain for the four species we performed stochastic simulation of the central dogma reactions (9), (10), and (11), using the central dogma rate constants in Table S1. The number of input levels, or values of *X* in [0,1] for the Gillespie simulations, was chosen based on the integration time. Hence, for higher integration times a larger set of protein distributions, P(g|X) was obtained from stochastic simulations to compute c(X;g). The number of input values *X* was selected by uniformly dividing the domain [0,1] into $2^{H\left(X\right)}$ intervals as reported in Table S2.

Gillespie simulations for each *X* were mainly performed for $10^{6}\;\min$ with the protein expression value sampled at an interval of 10min to obtain $10^{5}$ samples of the protein expression level *g*. Except for *M. musculus* and *H. sapiens* when 50<T≤200 the sampling interval was 100min, and when 500≤T the sampling interval was 200min.

The protein expression distributions P(g|X) were determined as the empirical distributions from the protein expression trajectory g(t). The number of bins $n_{b}$ used to construct the empirical distributions were chosen as $n_{b}=$ nearest integer larger than $2^{c_{\text{ideal}}\left(T\right)+\eta }$, where η>0, to use a larger number of bins than $2^{c_{\text{ideal}}\left(T\right)}$. The value of η for each species and integration time is in Table S3. For a more elaborate discussion on the selection of number of bins for computing channel capacity check.^4^^,^^12^^,^^27^

The protein expression trajectory from Gillespie simulations is an ergodic stationary process.^29^ As described above, the total duration of the sampled protein expression trajectory was between $10^{6}$ min to $2\times 10^{7}$ min depending on the species and the integration time. To test that we have captured the trajectory for a sufficient duration we performed a convergence analysis, by taking fractions of the protein expression trajectory data and computing the channel capacity. Specifically, we took the following fractions of the full trajectory data, 1/2,1/5,1/10,1/20,1/50 and 1/100.

Figure S8 shows the estimated protein-level channel capacity from the smaller trajectories along with the channel capacity from the full trajectory data. Smaller trajectories can overestimate the channel capacity, because the data from smaller trajectories overestimates the relative entropy between the conditional distributions P(g|X) for different values of *X*. For all the four species the estimated channel capacity from the “Full” and the ½ trajectory are indistinguishable, establishing convergence in the estimate. If the duration of a trajectory is too small to accurately estimate the conditional distributions, P(g|X), then doubling the trajectory length will produce a substantive difference in the estimated channel capacity. We observe this artifact of small trajectory data in Figure S8 when we compare the estimated channel capacity between the 1/50 and the 1/100 trajectories for *M. musculus* and *H. sapiens*.

##### Effect of fluctuation time period on information transfer

To determine how the time period of fluctuation of the input affects the information transfer from the input to the protein expression level, we determined the mutual information between the input and the ideal integration output $I\left(X;g_{\text{ideal}}\right)$ under fluctuating protocols of the input, *X* (Figure S9). We chose the same $\alpha ,l,k_{m}\text{,}$ and $k_{d,m}$ used in the simulation study for Figure S5, and selected two values of the protein decay rate constant $k_{d,g}=k_{d,m}/10$ and $k_{d,m}/100$, which has integration times T=10 and 100, respectively. For each of those two integration times we computed the ideal channel capacity $c_{\text{ideal}}\left(T\right)$ and the associated optimal input distribution, $P_{\text{opt}}\left(X\right)$ – the input distribution that achieves the channel capacity (Figure S10A). Then we considered a range of fluctuation time period for the input, $\tau_{X}=\frac{1}{k_{d,g}}\left\{0.1,0.2,0.5,1,2,5,10,20,50,100,200\right\}$. For each $\tau_{X}$ we performed a Gillespie simulation to capture the transcript trajectory m(t) when the input *X* fluctuates with time period $\tau_{X}$ assuming values according to the distribution $P_{\text{opt}}\left(X\right)$, the total duration of each simulation was $10^{4}\tau_{X}$. The stochastic transcript trajectory, m(t), was then convoluted with the integration kernel $e^{-k_{d,g}t}$ with $t\in \left[0,4/k_{d,g}\right]$, to obtain $g_{\text{ideal}}\left(t\right)$, which was subsequently used to compute the mutual information $I\left(X;g_{\text{ideal}}\right)$. An example of the stochastic trajectories under a fluctuating protocol of the input, *X*, is shown in Figure S9.

For the two integration times T=10 and 100, we obtained a set of ideal mutual information values, $I\left(X;g_{\text{ideal}}\right)$, as a function of the fluctuation time period of the input, shown in Figure S10B. We notice in Figure S10B, when the time period of fluctuation $\tau_{X}$ is larger than translation response time $1/k_{d,g}$, almost by a factor of 10, then the ideal mutual information value $I\left(X;g_{\text{ideal}}\right)$ approaches the ideal channel capacity for that integration time. When the fluctuation time period is smaller than $5/k_{d,g}$, then the ideal mutual information is less than half of the ideal channel capacity value. So, a relatively slow fluctuation in the environmental input is necessary to achieve the information gain possible due to integration of the transcript expression.

### Experimental model and subject details

The single-cell transcript and protein expression data for the inducible gene expression system were from a recently published manuscript by J.R.^22^ All experiments were performed with *E. coli* strain NEB 10-beta (New England Biolabs, MA, C3019) and the plasmids used in that work, pAN1201, pAN1717 and pAN1818 are already available on Addgene.

### Quantification and statistical analysis

Statistical testing was not used in this work.

## Data Availability

All original code has been deposited at https://github.com/sarkar-s/InCens.^65^ and is publicly available as of the date of publication. DOIs related to any other data sources are listed in the key resources table. Any additional information required to reanalyze the data reported in this paper is available from the lead contact upon request.

## References

[1] Crick F.H. (1958). On protein synthesis. Symp. Soc. Exp. Biol..

[2] Crick F. (1970). Central dogma of molecular biology. Nature.

[3] Cobb M. (2017). 60 years ago, francis crick changed the logic of biology. PLoS Biol..

[4] Cheong R., Rhee A., Wang C.J., Nemenman I., Levchenko A. (2011). Information transduction capacity of noisy biochemical signaling networks. Science.

[5] Tkačik G., Callan C.G., Bialek W. (2008). Information flow and optimization in transcriptional regulation. Proc. Natl. Acad. Sci. USA.

[6] So L.-h., Ghosh A., Zong C., Sepúlveda L.A., Segev R., Golding I. (2011). General properties of transcriptional time series in escherichia coli. Nat. Genet..

[7] Bowsher C.G., Swain P.S. (2012). Identifying sources of variation and the flow of information in biochemical networks. Proc. Natl. Acad. Sci. USA.

[8] Uda S., Saito T.H., Kudo T., Kokaji T., Tsuchiya T., Kubota H., Komori Y., Ozaki Y.-i., Kuroda S. (2013). Robustness and compensation of information transmission of signaling pathways. Science.

[9] Levchenko A., Nemenman I. (2014). Cellular noise and information transmission. Curr. Opin. Biotechnol..

[10] Selimkhanov J., Taylor B., Yao J., Pilko A., Albeck J., Hoffmann A., Tsimring L., Wollman R. (2014). Accurate information transmission through dynamic biochemical signaling networks. Science.

[11] Komorowski M., Tawfik D.S. (2019). The limited information capacity of cross-reactive sensors drives the evolutionary expansion of signaling. Cell Syst..

[12] Suderman R., Bachman J.A., Smith A., Sorger P.K., Deeds E.J. (2017). Fundamental trade-offs between information flow in single cells and cellular populations. Proc. Natl. Acad. Sci. USA.

[13] Mundt M., Anders A., Murray S.M., Sourjik V. (2018). A system for gene expression noise control in yeast. ACS Synth. Biol..

[14] Tabbaa O.P., Jayaprakash C. (2014). Mutual information and the fidelity of response of gene regulatory models. Phys. Biol..

[15] Hansen A.S., O’Shea E.K. (2015). Limits on information transduction through amplitude and frequency regulation of transcription factor activity. Elife.

[16] Mc Mahon S.S., Sim A., Filippi S., Johnson R., Liepe J., Smith D., Stumpf M.P. (2014). Information theory and signal transduction systems: from molecular information processing to network inference. Semin. Cell Dev. Biol..

[17] Lan G., Tu Y. (2016). Information processing in bacteria: memory, computation, and statistical physics: a key issues review. Rep. Prog. Phys..

[18] Granados A.A., Pietsch J.M.J., Cepeda-Humerez S.A., Farquhar I.L., Tkačik G., Swain P.S. (2018). Distributed and dynamic intracellular organization of extracellular information. Proc. Natl. Acad. Sci. USA.

[19] Patange S., Girvan M., Larson D.R. (2018). Single-cell systems biology: probing the basic unit of information flow. Curr. Opin. Struct. Biol..

[20] Hausser J., Mayo A., Keren L., Alon U. (2019). Central dogma rates and the trade-off between precision and economy in gene expression. Nat. Commun..

[21] Cover T.M. (1999).

[22] Rammohan J., Lund S., Alperovich N., Paralanov V., Strychalski E., Ross D. (2021). Comparison of bias and resolvability in single-cell and single-transcript methods. Commun. Biol..

[23] Rammohan J. (2020). Results of Single-Cell and Single-Transcript Measurements Collected for Bias and Resolvability Attribution Using Split Samples (Brass) Study.

[24] Bialek W. (2012).

[25] Blahut R. (1972). Computation of channel capacity and rate-distortion functions. IEEE Trans. Inf. Theor..

[26] Yeung R.W. (2008).

[27] Sarkar S., Tack D., Ross D. (2020). Sparse estimation of mutual information landscapes quantifies information transmission through cellular biochemical reaction networks. Commun. Biol..

[28] Rammohan J., Sarkar S., Ross D. (2022). Single-cell measurement quality in bits. PLOS One accepted.

[29] Stamatakis M., Mantzaris N.V. (2009). Comparison of deterministic and stochastic models of the lac operon genetic network. Biophys. J..

[30] Li G.-W., Xie X.S. (2011). Central dogma at the single-molecule level in living cells. Nature.

[31] Kabanov Y.M. (1978). The capacity of a channel of the Poisson type. Theory Probab. Appl..

[32] Lapidoth A., Moser S.M. (2009). On the capacity of the discrete-time Poisson channel. IEEE Trans. Inf. Theor..

[33] Silverman R. (1955). On binary channels and their cascades. IEEE Trans. Inf. Theor..

[34] Kiely A.B., Coffey J.T. (1993). On the capacity of a cascade of channels. IEEE Trans. Inf. Theor..

[35] Niesen U., Fragouli C., Tuninetti D. (2007). On capacity of line networks. IEEE Trans. Inf. Theor..

[36] Bernstein J.A., Khodursky A.B., Lin P.-H., Lin-Chao S., Cohen S.N. (2002). Global analysis of mrna decay and abundance in escherichia coli at single-gene resolution using two-color fluorescent dna microarrays. Proc. Natl. Acad. Sci. USA.

[37] Nagar N., Ecker N., Loewenthal G., Avram O., Ben-Meir D., Biran D., Ron E., Pupko T. (2021). Harnessing machine learning to unravel protein degradation in escherichia coli. mSystems.

[38] Eser P., Demel C., Maier K.C., Schwalb B., Pirkl N., Martin D.E., Cramer P., Tresch A. (2014). Periodic mrna synthesis and degradation co-operate during cell cycle gene expression. Mol. Syst. Biol..

[39] Belle A., Tanay A., Bitincka L., Shamir R., O’Shea E.K. (2006). Quantification of protein half-lives in the budding yeast proteome. Proc. Natl. Acad. Sci. USA.

[40] Schwanhäusser B., Busse D., Li N., Dittmar G., Schuchhardt J., Wolf J., Chen W., Selbach M. (2011). Global quantification of mammalian gene expression control. Nature.

[41] Friedel C.C., Dölken L., Ruzsics Z., Koszinowski U.H., Zimmer R. (2009). Conserved principles of mammalian transcriptional regulation revealed by rna half-life. Nucleic Acids Res..

[42] Cambridge S.B., Gnad F., Nguyen C., Bermejo J.L., Krüger M., Mann M. (2011). Systems-wide proteomic analysis in mammalian cells reveals conserved, functional protein turnover. J. Proteome Res..

[43] Paulsson J. (2004). Summing up the noise in gene networks. Nature.

[44] Paulsson J. (2005). Models of stochastic gene expression. Phys. Life Rev..

[45] McGrath T., Jones N.S., Ten Wolde P.R., Ouldridge T.E. (2017). Biochemical machines for the interconversion of mutual information and work. Phys. Rev. Lett..

[46] Peccoud J., Ycart B. (1995). Markovian modeling of gene-product synthesis. Theor. Popul. Biol..

[47] Pilkiewicz K.R., Mayo M.L. (2016). Fluctuation sensitivity of a transcriptional signaling cascade. Phys. Rev. E.

[48] Rowland M.A., Pilkiewicz K.R., Mayo M.L. (2021). Devil in the details: mechanistic variations impact information transfer across models of transcriptional cascades. PLoS One.

[49] Ghusinga K.R., Dennehy J.J., Singh A. (2017). First-passage time approach to controlling noise in the timing of intracellular events. Proc. Natl. Acad. Sci. USA.

[50] Thomas P. (2017). Making sense of snapshot data: ergodic principle for clonal cell populations. J. R. Soc. Interface.

[51] Hambraeus G., von Wachenfeldt C., Hederstedt L. (2003). Genome-wide survey of mrna half-lives in bacillus subtilis identifies extremely stable mrnas. Mol. Genet. Genom..

[52] Nordholt N., van Heerden J.H., Bruggeman F.J. (2020). Biphasic cell-size and growth-rate homeostasis by single bacillus subtilis cells. Curr. Biol..

[53] Ozarow L.H., Shamai S., Wyner A.D. (1994). Information theoretic considerations for cellular mobile radio. IEEE Trans. Veh. Technol..

[54] Lapidoth A., Moser S.M. (2003). Capacity bounds via duality with applications to multiple-antenna systems on flat-fading channels. IEEE Trans. Inf. Theor..

[55] Kafri M., Metzl-Raz E., Jona G., Barkai N. (2016). The cost of protein production. Cell Rep..

[56] Dekel E., Alon U. (2005). Optimality and evolutionary tuning of the expression level of a protein. Nature.

[57] Scott M., Gunderson C.W., Mateescu E.M., Zhang Z., Hwa T. (2010). Interdependence of cell growth and gene expression: origins and consequences. Science.

[58] Lan G., Sartori P., Neumann S., Sourjik V., Tu Y. (2012). The energy–speed–accuracy trade-off in sensory adaptation. Nat. Phys..

[59] Kalisky T., Dekel E., Alon U. (2007). Cost–benefit theory and optimal design of gene regulation functions. Phys. Biol..

[60] Parrondo J.M.R., Horowitz J.M., Sagawa T. (2015). Thermodynamics of information. Nat. Phys..

[61] De Martino D., Capuani F., De Martino A. (2017). Quantifying the entropic cost of cellular growth control. Phys. Rev. E.

[62] Lambert G., Kussell E. (2014). Memory and fitness optimization of bacteria under fluctuating environments. PLoS Genet..

[63] Schwartz A., Gaigalas A.K., Wang L., Marti G.E., Vogt R.F., Fernandez-Repollet E. (2004). Formalization of the mesf unit of fluorescence intensity. Cytometry B Clin. Cytom..

[64] Sarkar S. (2020). InGene, github.

[65] Sarkar S. (2023). InCens, github.

[66] Smirnova G.V., Oktyabrsky O.N. (2018). Relationship between escherichia coli growth rate and bacterial susceptibility to ciprofloxacin. FEMS Microbiol. Lett..

