# Supplementary material for: Nearly maximal information gain due to time integration in central dogma reactions
Source: iScience. 2023 Apr 28;26(6):106767. doi: 10.1016/j.isci.2023.106767 (PMC10206154; doi:10.1016/j.isci.2023.106767)
Supplement: Document S1. Figures S1–S10 and Tables S1–S3 [file mmc1.pdf]

iScience, Volume *26*

Supplemental information

Nearly maximal information gain due to time integration in central dogma reactions

Swarnavo Sarkar and Jayan Rammohan

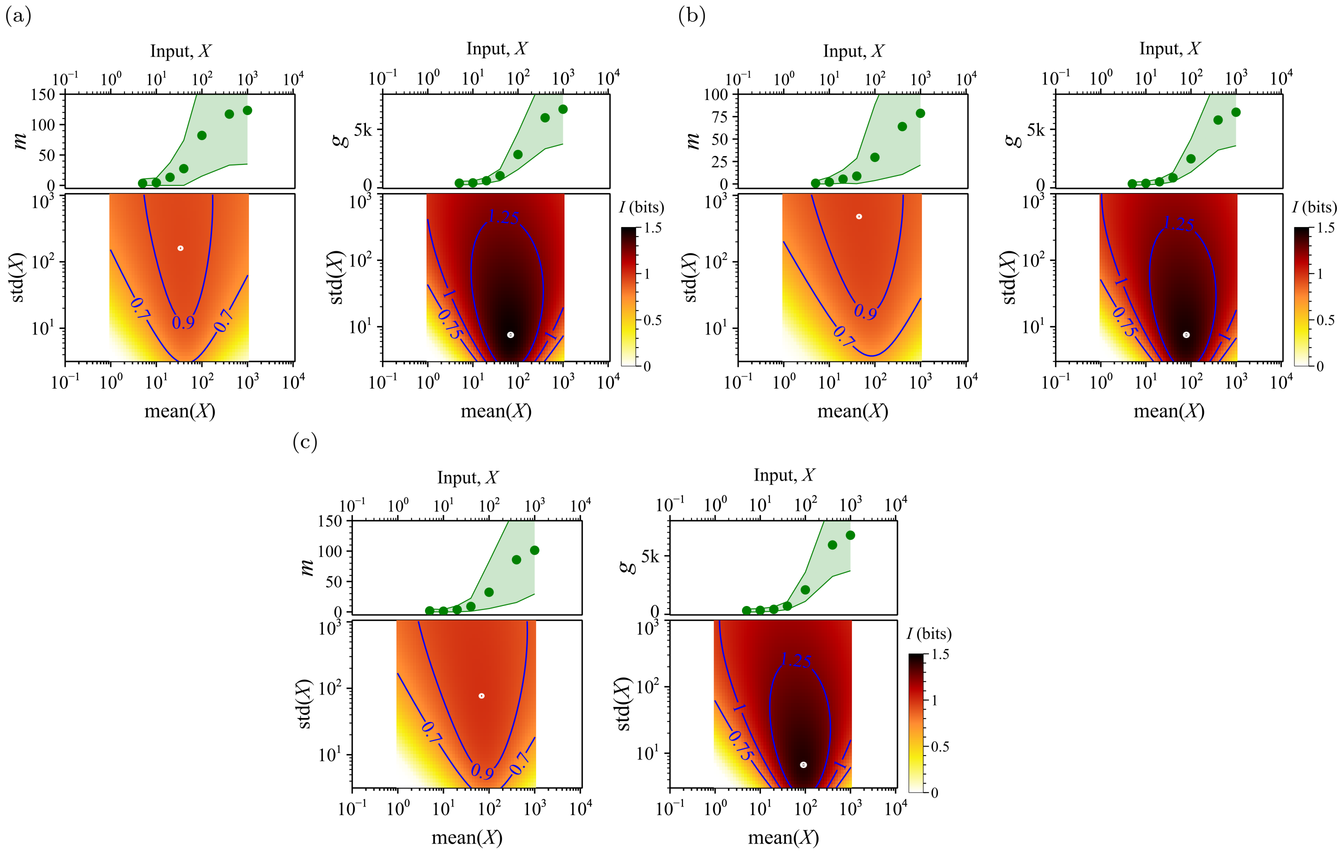

Figure S1: Transcript and protein-level mutual information landscapes for the three biological replicates measured experimentally: (a) replicate 1, (b) replicate 2, and (c) replicate 3. The transcript and the protein-level dose response curves (in green) and the mutual information landscapes (heat map) have the same annotation as in Figure 1C. Related to Figure 1C.

Biological replicates of the mutual information landscapes reported in Figure 1C. The experimental method for measuring the biological replicates are reported in [22].

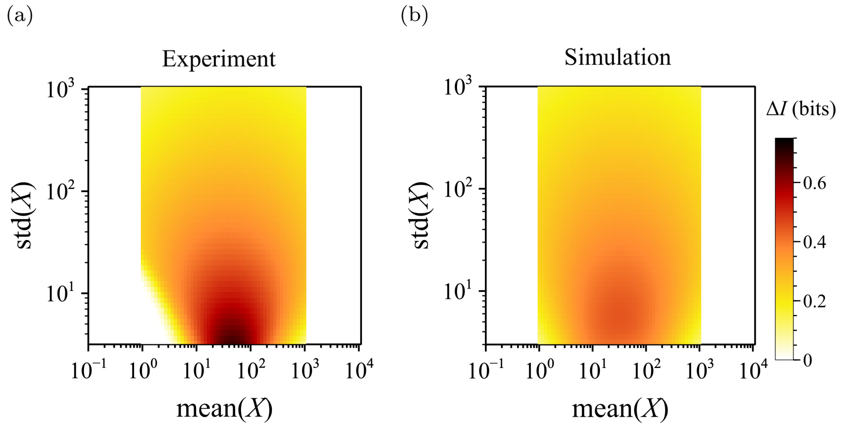

Figure S2: Difference between the protein-level and the transcript-level mutual information, $\Delta I = I(X;g) - I(X;m)$, from both the experimental and the simulated data. The difference in mutual information values are shown over the space of probability distributions of the input. Input distributions, $P(X)$, are identified in the landscape using the mean and the standard deviation as a two dimensional coordinate. (a) Gain in the protein-level mutual information, $I(X;g)$, compared to the transcript-level mutual information, $I(X;m)$, for the experimental inducible gene expression system shown in Figure 1(a) of the manuscript. (b) Gain in the protein-level mutual information, $I(X;g)$, compared to the transcript-level mutual information, $I(X;m)$, for the simulated inducible gene expression system [27]. Related to Figures 1C and 1D.

Difference in the protein-level and the transcript-level mutual information from the experimental and the simulation data, which were reported in Figures 1C and 1D.

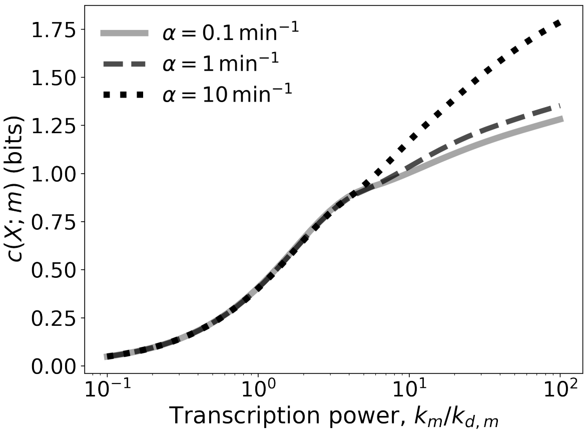

Figure S3: Rate of increase in the transcript-level channel capacity depending on the relative value of the frequency parameter α with respect to transcript decay rate constant. $k_{d,m} = 0.5\,\mathrm{min}^{-1}$ for all cases. We used 16 uniformly spaced values of the input $X \in [0, 1]$, to determine k_{ON}, k_{OFF} from Eq. (12), with leakiness $l = 0.01$, for all cases. The transcript expression distributions, $P(m|X)$, were obtained as Negative Binomial distributions as a function of k_{ON}, K_{OFF}, k_m, and $k_{d,m}$ [46, 6]. Related to Figure 2A.

Transition in the rate of growth of transcript-level channel capacity, observed in Figure 2A, is due to the relative value of the frequency parameter as shown in Figure S3.

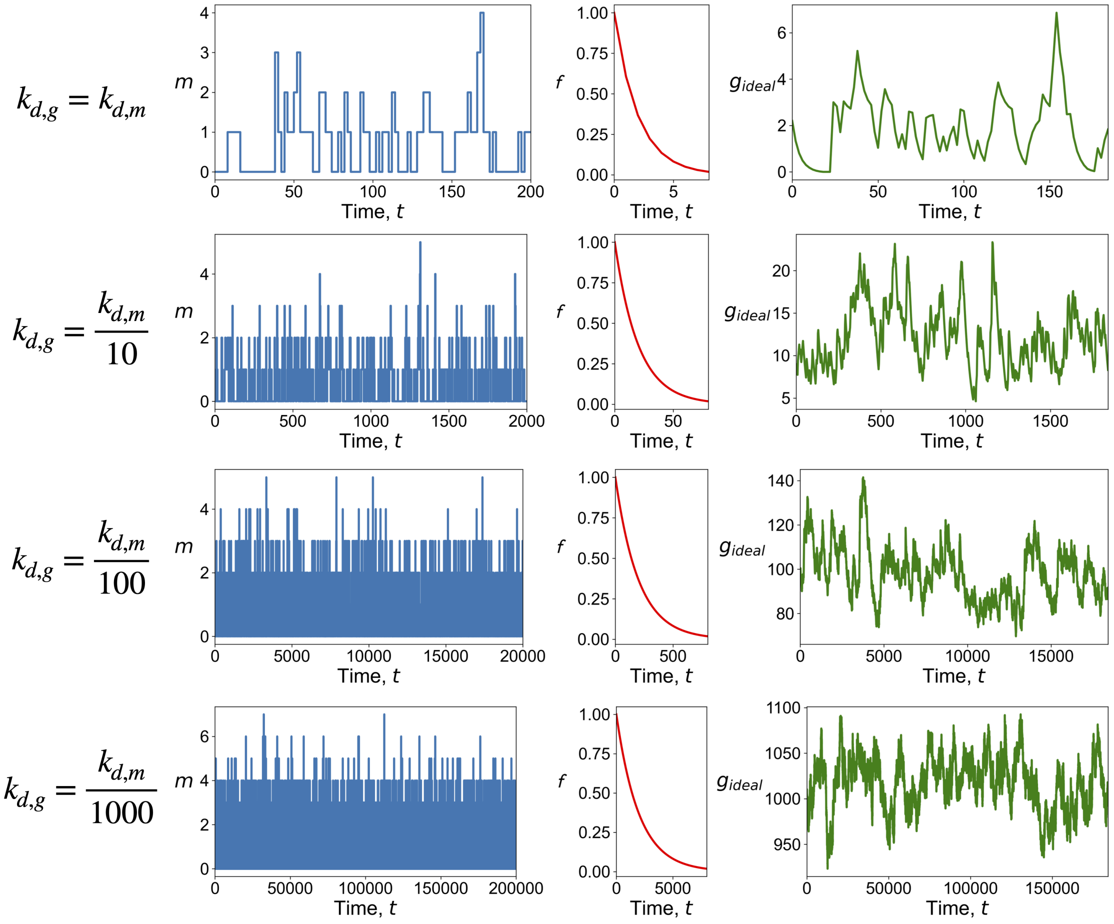

Figure S4: Integration of the stochastic time-dependent transcript expression level as a function of the protein decay rate constant $k_{d,g}$. The transcript decay rate constant is $k_{d,m} = 0.5\,\text{min}^{-1}$ for all the examples, and the protein decay rate constant, $k_{d,g}$, is a decreasing fraction of the $k_{d,m}$ for each row of the figure. The stochastic transcript expression, $m(t)$, was captured for a duration of the $100/k_{d,g}$, sampled at interval of $1/k_{d,m}$. The convolution kernel function f has the domain $[0, 4/k_{d,g}]$, with values at an interval of $1/k_{d,m}$. The output of the convolution $g_{\text{ideal}}(t)$ is shown in green. The average g_{ideal} value increases as a function of increasing response time for translation, $1/k_{d,g}$, which is due to the increasing integration of the transcript expression level. Related to Figure 3A and STAR Methods: Deterministic integration approximation for the translation output.

The maximum possible information gain during translation, shown in Figure 3A, occurs because of time integration of the transcript expression trajectory demonstrated in Figure S4.

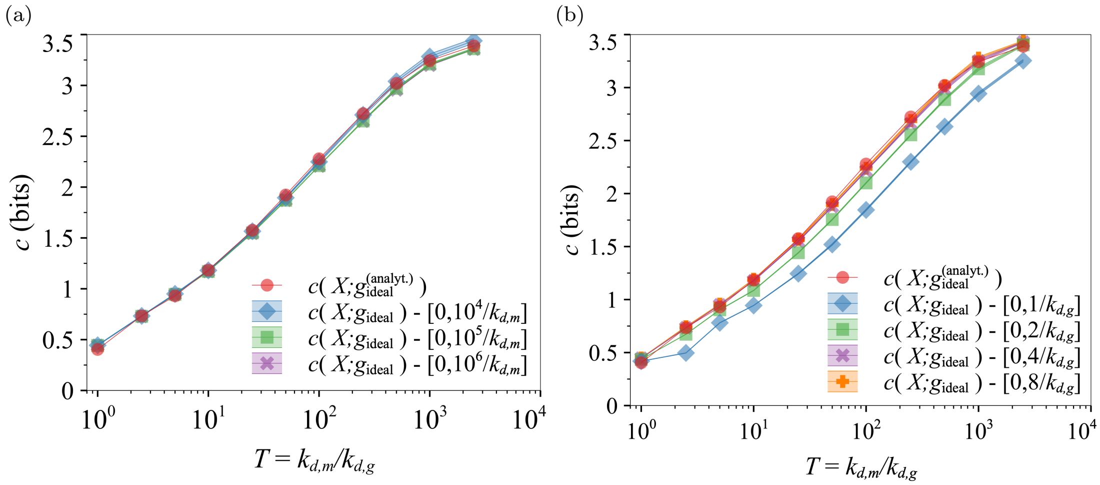

Figure S5: Estimate of the information gain due to integration using the numerical and analytical methods as described in STAR Methods. (a) Effect of sample size or the length of the transcript expression trajectory $m(t)$ on the estimate of the information gain curve $c(X; g_{\text{ideal}})$-vs-T. The 95% confidence interval for each channel capacities $c(X; g_{\text{ideal}})$ was obtained from 20 replicates. (b) Effect of the time domain size for the kernel function $f(t)$ on the estimate of the information gain curve $c(X; g_{\text{ideal}})$-vs-T. The 95% confidence interval for each channel capacities $c(X; g_{\text{ideal}})$ was obtained from 10 replicates. In both (a) and (b) the red solid line with dots show the ideal information gain using the analytical approximation to the ideal integration output, Eq. (27). Related to Figures 3A and STAR Methods: Validation of the analytical approximation for ideal channel capacity.

Validation of the analytical approximation of the ideal integration output, g_{ideal}, which is used to compute the ideal channel capacity in Figure 3A.

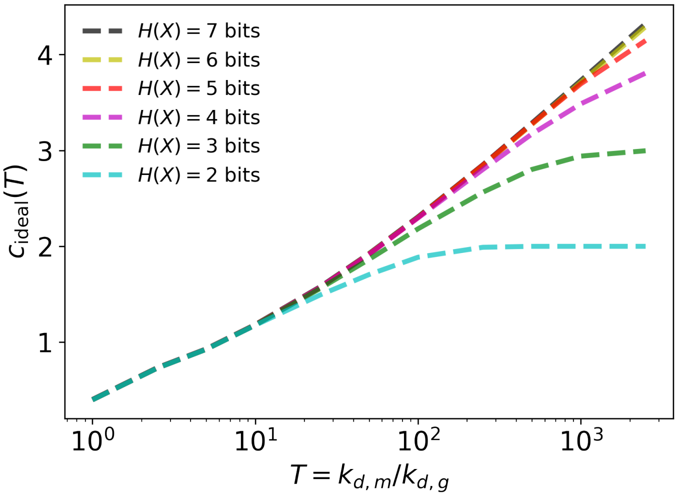

Figure S6: Ideal information gain $c_{\mathrm{ideal}}(T)$ for increasing number of input values. Each ideal information gain curve was computed from $P(g_{\mathrm{ideal}}^{(\mathrm{analyt.})}|X)$ for $2^{H(X)}$ uniformly-spaced values of the input $X \in [0, 1]$. For a fixed integration time, *e.g.*, $T = 1000$, the estimated ideal channel capacity initially increases with $H(X)$, but eventually converges to a value that is independent of $H(X)$. Related to Figures 3A, 3B and 3D and STAR Methods: Effect of number of input levels on the estimate of $c_{\mathrm{ideal}}(T)$.

The ideal channel capacity evaluated in Figures 3A, 3B, and 3D, can be underestimated due to the number of levels of the input, X. Figure SI 6 shows how to avoid underestimation of $c_{\mathrm{ideal}}(T)$ by systematically increasing the number of input levels.

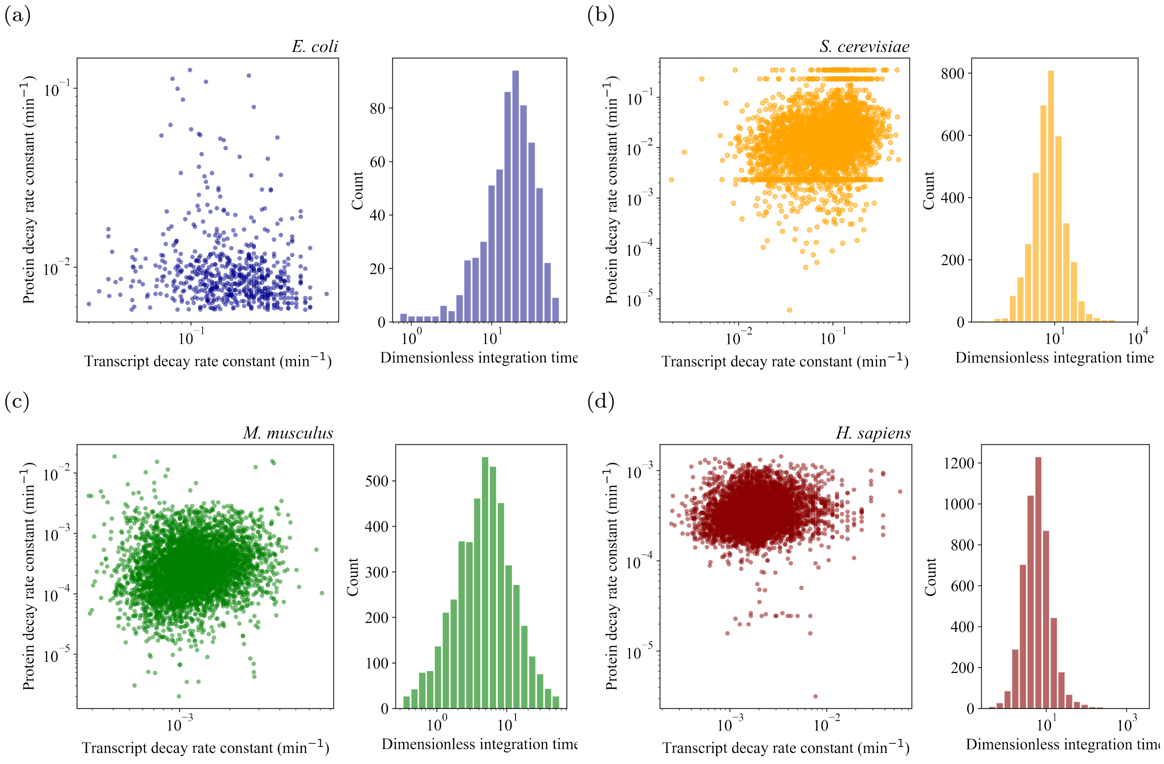

Figure S7: Paired transcript and protein decay rates and the distribution of dimensionless integration times for the four species. (a) Integration time distribution for *E. coli*. The effective protein decay rate was determined using a doubling time of 2 h [66]. (b) Integration time distribution for *S. cerevisiae*. (c) Integration time distribution for *M. musculus*. (d) Integration time distribution for *H. sapiens*. Related to Figure 3C and Key Resources Table.

Paired transcript and protein decay rate constants for the four species obtained from the sources reported in the Key Resources Table. The paired decay rate constants are used to construct the integration time distributions reported in Figure 3C.

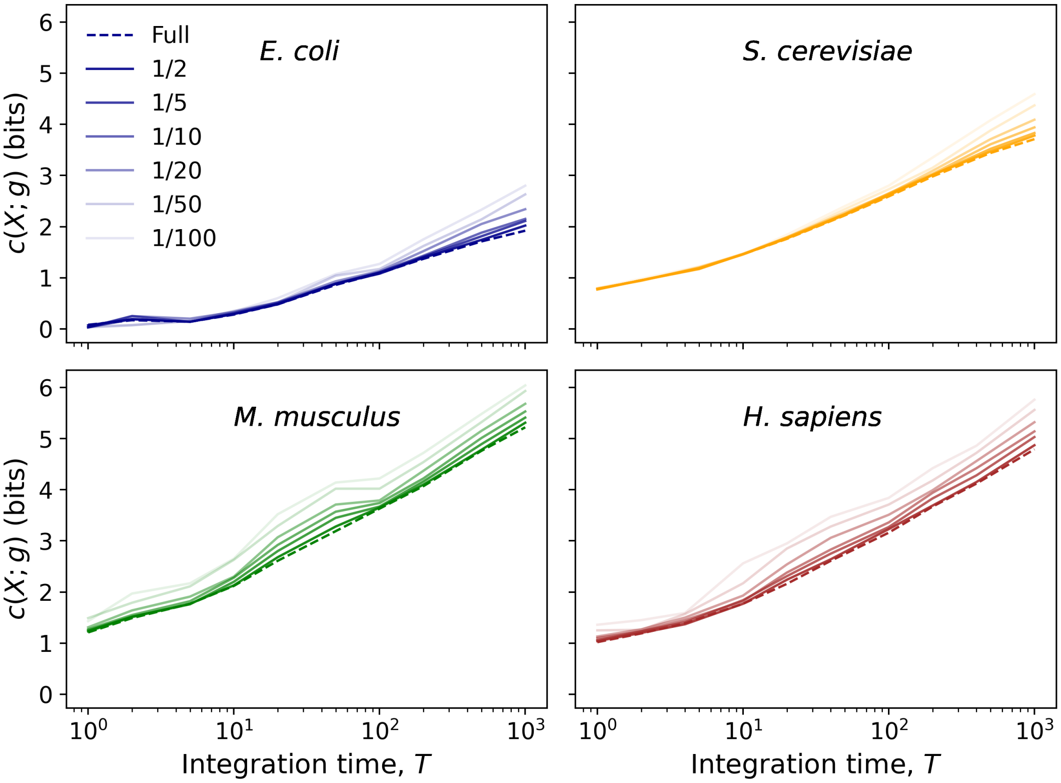

Figure S8: Estimated protein-level channel capacity using smaller protein expression trajectories. The channel capacity values estimated from the "Full" trajectory are the values used in Figure 3D in the main text. Estimated channel capacity from smaller trajectories are shown in lighter shades. The labels in the legend specifies the fraction of the full trajectory that was used to estimate the conditional protein expression distribution, $P(g|X)$, and then the channel capacity, $c(X; g)$. Related to Figure 3D and STAR Methods: Stochastic simulations of central dogma master equations.

The estimated protein-level channel capacity for each species, reported in Figure 3D, can depend on the number of samples of the protein expression level obtained from the Gillespie simulations. Figure S7 shows that the stochastic simulation trajectories are sufficiently long so that the estimated $c(X; g)$ are independent of the number of samples.

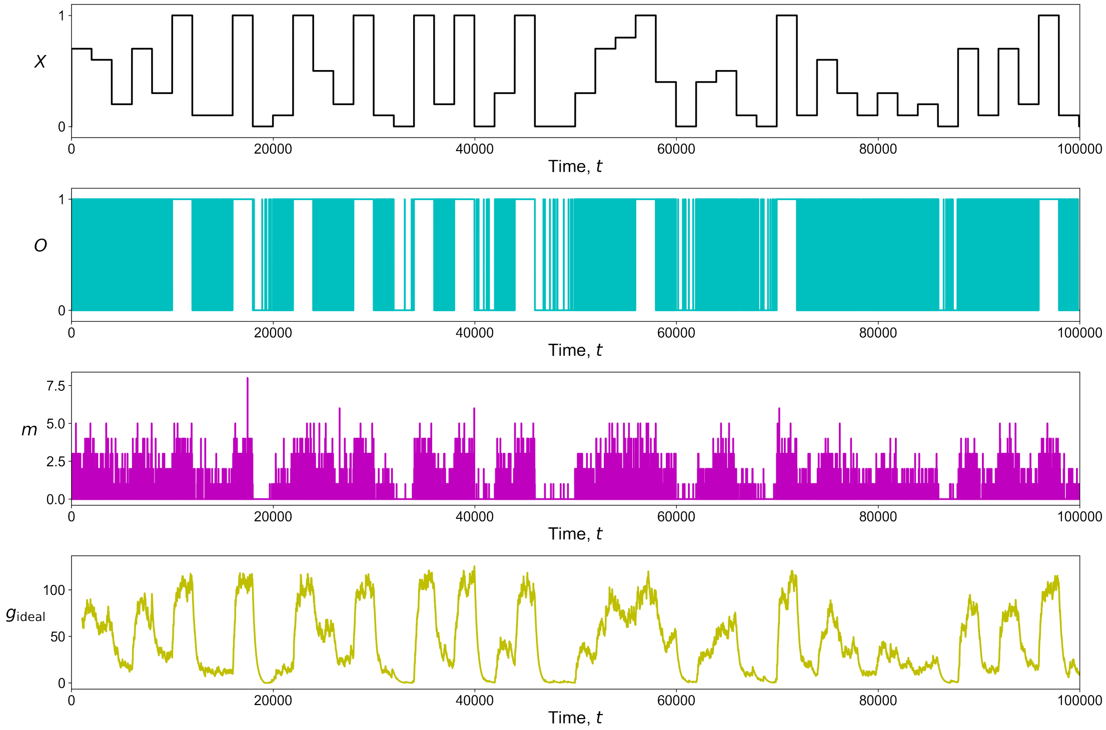

Figure S9: An example stochastic trajectory with the operator state O, transcript expression level m, and the ideal integration output g_{ideal}, under a fluctuating protocol of the input, X. The input X is changed stochastically after an interval of τ_X. Related to Figure 3D and STAR Methods: Effect of fluctuation time period on information transfer.

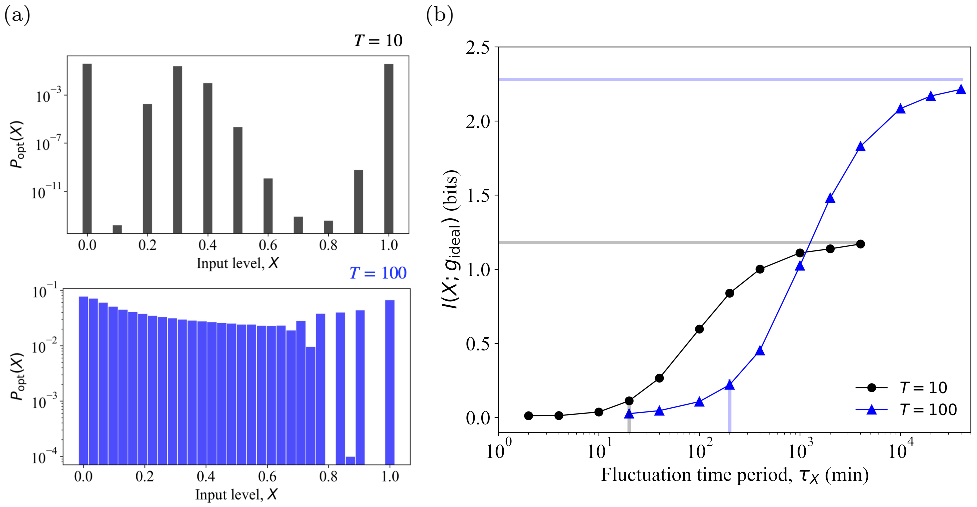

Figure S10: Ideal protein-level mutual information under a fluctuating protocol of the input, with fluctuation time period τ_X. (a) Optimal input distribution, $P_{\text{opt}}(X)$, that achieves the ideal channel capacity, $c_{\text{ideal}}(T)$, for integration times of 10 and 100. (b) Mutual information between the input X and the ideal integration output g_{ideal} for fluctuating protocols with the same input distribution $P_{\text{opt}}(X)$ but with different fluctuation time periods, τ_X. The horizontal translucent lines represent the channel capacity $c_{\text{ideal}}(T)$. $k_{d,m} = 0.5\,\text{min}^{-1}$ for both the cases, so $k_{d,g} = 0.05\,\text{min}^{-1}$ for $T = 10$ and $k_{d,g} = 0.005\,\text{min}^{-1}$ for $T = 100$, respectively. The vertical translucent lines represent the translation response times $1/k_{d,g}$. Related to Figure 3D and STAR Methods: Effect of fluctuation time period on information transfer.

Relatively fast fluctuations in the environmental input decreases the protein-level information transfer to below capacity, which can explain the existing range of integration times for the four species, shown in Figure 3D.

species	k_m (min^{-1})	$k_{d,m}$ (min^{-1})	k_g (min^{-1})	$k_{d,g}$ (min^{-1})
E. coli	0.02	0.15	$\{18, 9, 4.5, 1.8\}$	$\{0.03, 0.015, 0.0075, 0.003\}$
S. cerevisiae	0.31	0.1	$\{40, 20, 8, 4, 2, 0.8\}$	$\{0.1, 0.05, 0.02, 0.01, 0.005, 0.002\}$
M. musculus	0.02	0.002	$\{50, 20, 10, 5, 2\}$	$\{0.002, 0.001, 0.0004, 0.0002, 0.0001\}$
H. sapiens	0.01	0.002	$\{20, 10, 4, 2\}$	$\{0.001, 0.0005, 0.0002, 0.0001\}$

Table S1: Central dogma rate constants used to compute the typical protein-level information gain curves for the four species, solid lines with markers in Figure 3D. The translation loss region in Figure 3D was computed from the ideal and the protein level information gain curves for integration time range $T \in [1, 1000]$. Related to Figure 3D.

species	Integration time range	$H(X)$
E. coli and *S. cerevisiae*	$T \leq 10$	4
	$10 < T \leq 200$	5
	$200 < T \leq 1000$	6
M. musculus and *H. sapiens*	$T \leq 10$	4
	$10 < T \leq 40$	5
	$40 < T \leq 200$	6
	$200 < T \leq 1000$	7

Table S2: Number of input levels to obtain the conditional protein expression distribution $P(g|X)$ from stochastic simulation. The domain $[0,1]$ was uniformly divided into $2^{H(X)}$ values to get the set of input values X for each species and integration time range. Related to Figure 3D and STAR Methods: Stochastic simulations of central dogma master equations.

species	Ideal channel capacity	η
	$c_{\text{ideal}}(T) \leq 0.1$	2.5
E. coli	$0.1 < c_{\text{ideal}}(T) \leq 1.0$	3.0
	$2.0 < c_{\text{ideal}}(T) \leq 1.5$	2.0
	$1.5 < c_{\text{ideal}}(T)$	1.5
S. cerevisiae,	$c_{\text{ideal}}(T) \leq 1.0$	2.0
M. musculus	$1.0 < c_{\text{ideal}}(T) \leq 2.0$	1.5
and *H. sapiens*	$2.0 < c_{\text{ideal}}(T)$	1.0

Table S3: Number of bins to construct the conditional distributions, $P(g|X)$, using 10^5 samples from the protein expression level trajectory, $g(t)$. The number of bins for each species and integration time is the nearest integer larger than $2^{c_{\text{ideal}}(T)+\eta}$. Related to Figure 3D and STAR Methods: Stochastic simulations of central dogma master equations.
